# Smoking-Associated DNA Methylation Biomarkers and Their Predictive Value for All-Cause and Cardiovascular Mortality

**DOI:** 10.1289/ehp.1409020

**Published:** 2015-05-27

**Authors:** Yan Zhang, Ben Schöttker, Ines Florath, Christian Stock, Katja Butterbach, Bernd Holleczek, Ute Mons, Hermann Brenner

**Affiliations:** 1 Division of Clinical Epidemiology and Aging Research, German Cancer Research Center, Heidelberg, Germany; 2 Institute of Medical Biometry and Informatics, University of Heidelberg, Heidelberg, Germany; 3 Saarland Cancer Registry, Saarbrücken, Germany

## Abstract

**Background:**

With epigenome-wide mapping of DNA methylation, a number of novel smoking-associated loci have been identified.

**Objectives:**

We aimed to assess dose–response relationships of methylation at the top hits from the epigenome-wide methylation studies with smoking exposure as well as with total and cause-specific mortality.

**Methods:**

In a population-based prospective cohort study in Germany, methylation was quantified in baseline blood DNA of 1,000 older adults by the Illumina 450K assay. Deaths were recorded during a median follow-up of 10.3 years. Dose–response relationships of smoking exposure with methylation at nine CpGs were modeled by restricted cubic spline regression. Associations of individual and aggregate methylation patterns with all-cause, cardiovascular, and cancer mortality were assessed by multiple Cox regression.

**Results:**

Clear dose–response relationships with respect to current and lifetime smoking intensity were consistently observed for methylation at six of the nine CpGs. Seven of the nine CpGs were also associated with mortality outcomes to various extents. A methylation score based on the top two CpGs (cg05575921 and cg06126421) showed the strongest associations with all-cause, cardiovascular, and cancer mortality, with adjusted hazard ratios (95% CI) of 3.59 (2.10, 6.16), 7.41 (2.81, 19.54), and 2.48 (1.01, 6.08), respectively, for participants with methylation levels in the lowest quartile at both CpGs. Adding methylation at those two CpGs into a model that included the variables of the Systematic Coronary Risk Evaluation chart for fatal cardiovascular risk prediction improved the predictive discrimination.

**Conclusion:**

The novel methylation biomarkers are highly informative for both smoking exposure and smoking-related mortality outcomes. In particular, these biomarkers may substantially improve cardiovascular risk prediction. Nevertheless, the findings of the present study need to be further validated in additional large longitudinal studies.

**Citation:**

Zhang Y, Schöttker B, Florath I, Stock C, Butterbach K, Holleczek B, Mons U, Brenner H. 2016. Smoking-associated DNA methylation biomarkers and their predictive value for all-cause and cardiovascular mortality. Environ Health Perspect 124:67–74; http://dx.doi.org/10.1289/ehp.1409020

## Introduction

Tobacco smoking has been recognized as a risk factor for a variety of complex diseases (CDC 2014), including cardiovascular diseases (CVDs) (Ezzati et al. 2005b), at least 15 types of cancer (Ezzati et al. 2005a), and pulmonary diseases (Decramer et al. 2012). Nevertheless, accurate prediction of smoking-attributable health risk is still hampered by various factors (CDC 2010). In particular, it is well known that self-reported smoking exposure suffers from recall bias or intentional underreporting (Connor Gorber et al. 2009; Rebagliato 2002). Even though a number of biomarkers are well established, such as breath carbon monoxide (CO) and cotinine levels, they exclusively reflect short-term smoking exposure and are of limited use for quantifying cumulative exposure and consequently for predicting smoking-related risk (CDC 2010). DNA or protein adducts are considered integrative biomarkers that reflect internal effective dose of smoking, which may, however, only be useful for carcinogenic risk assessment (CDC 2010; Lodovici and Bigagli 2009). In cardiovascular risk assessment, although several biomarkers have been described and used, no biomarker has yet been identified for specifically predicting smoking-related risk (CDC 2010).

Recent advances in genome-wide methylation profiling have opened new avenues in the search for biomarkers reflecting both current and lifetime smoking exposure that might have the potential to enhance prediction of smoking-related risks. Recently, a number of novel smoking-associated blood DNA methylation biomarkers were identified by using the Infinium HumanMethylation Illumina 450K BeadChip (Joubert et al. 2012; Shenker et al. 2013a; Zeilinger et al. 2013), among which seven loci located in four intragenic or intergenic regions [including *F2RL3* (cg03636183), *AHRR* (cg21161138 and cg05575921), *2q37.1* (cg21566642, cg01940273, and cg05951221), *6p21.33* (cg06126421)] were the top seven CpGs reported by both epigenome-wide studies conducted in adults (Shenker et al. 2013a; Zeilinger et al. 2013). To further explore the use of methylation levels of these regions for quantifying biologically effective smoking exposure and for enhancing risk prediction of smoking-related disease, we carried out comprehensive analyses on the associations of methylation at nine CpGs [the top seven CpGs listed above and two other CpGs [*AHRR* (cg23576855); *2q37.1* (cg06644428)] in those regions reported to be associated with smoking (Shenker et al. 2013a; Zeilinger et al. 2013)] with both current and lifetime smoking exposure as well as mortality in a population-based cohort of older adults. In addition, we aimed to evaluate whether these methylation biomarkers can improve the fatal cardiovascular risk prediction estimated by the Systematic Coronary Risk Evaluation (SCORE) chart of the European Society of Cardiology (Conroy et al. 2003).

## Methods

*Study design and data collection*. The study subjects were selected from the ESTHER study, a statewide population-based cohort study conducted in southwest Germany (Schöttker et al. 2013a). Briefly, 9,949 older adults (50–75 years of age) were enrolled by their general practitioners during a routine health check-up between July 2000 and December 2002, and followed up since then. The distribution of sociodemographic factors and major risk factors in the cohort was similar to the distribution seen in representative surveys of the population in Germany in the corresponding age range (Löw et al. 2004). A genome-wide methylation screen was performed in baseline blood samples of 1,000 participants who were recruited between July and October 2000 (i.e., those with the longest follow-up time) and included in the current analysis. The study was approved by the ethics committees of the University of Heidelberg and of the state medical board of Saarland, Germany. Written informed consent was obtained from all participants.

Participants’ sociodemographic characteristics, lifestyle factors, health status, and history of major diseases at baseline were obtained by a standardized self-administered questionnaire. Detailed information on lifetime active smoking was also ascertained from the self-administered questionnaire, including age at initiation of smoking and intensity of smoking at various ages, as well as age of smoking cessation for former smokers. Additional information on height, weight, blood pressure, and prevalent diseases (e.g., diabetes, hypertension, CVD) was extracted from a standardized form completed by the general practitioners during the health check-ups. Prevalent CVD at baseline was defined by either physician-reported coronary heart disease or a self-reported history of myocardial infarction, stroke, pulmonary embolism, or revascularization of coronary arteries. Prevalent cancer [*International Classification of Diseases, 10th Revision* (ICD-10) codes C00–C99 except nonmelanoma skin cancer (code C44)] was determined by self-report or record linkage with data from the Saarland Cancer Registry [http://www.krebsregister.saarland.de/ziele/ziel1.html (in German)]. Blood samples (21 mL from each participant) were collected during the health check-up and aliquoted and stored at –80°C until further processing. Total cholesterol level was measured in serum by standard high-performance liquid chromatography methods (Schöttker et al. 2013b). Deaths during follow-up (between 2000 and end of 2011) were identified by record linkage with population registries in Saarland; few participants who moved out of Saarland were censored at the date last known to be alive. Information about the major cause of death was obtained from death certificates provided by the local public health offices, and were coded with ICD-10 codes. Cardiovascular and cancer deaths were defined by ICD-10 codes I00–I99 and C00–C99, respectively; nonmelanoma skin cancer (ICD-10 code C44) was excluded.

*Methylation assessment*. DNA was extracted from whole blood samples collected at baseline by a salting out procedure (Miller et al. 1988) and was allocated in the 96-well format. Three random duplicate samples were placed on each plate as quality controls. The Infinium HumanMethylation450K BeadChip Assay (Illumina Inc., San Diego, CA, USA) was used to quantify DNA methylation at 485,577 CpG sites. Briefly, a sample of 1.5 μg genomic DNA was bisulfite converted, and 200 ng bisulfite-treated DNA was applied to the 450K BeadChips. The samples were analyzed following the manufacturer’s instruction at the Genomics and Proteomics Core Facility of German Cancer Research Center. GenomeStudio® (version 2011.1; Illumina Inc.) was used to extract DNA methylation signals from the scanned arrays (module version 1.9.0; Illumina Inc.) and to calculate methylation intensity (β-value) as a ratio of the methylated signal over the sum of the methylated and unmethylated signals at each CpG according to the manufacturer’s guide, without additional background correction. Data were normalized to internal controls provided by Illumina (Illumina normalization). Methylation intensities at the nine CpGs were extracted from the 450K data.

*Statistical analysis*. Median methylation intensities at the nine CpGs were determined for strata of sociodemographic characteristics, lifestyle factors, and prevalent diseases; differences in methylation intensities between strata were examined by Kruskal–Wallis tests. Correlations between methylation intensity at the nine CpGs were assessed by Spearman rank correlation coefficients. The associations between smoking indicators (including smoking status, current intensity of smoking, cumulative dose of smoking, and time since cessation of smoking) and methylation intensity at the nine CpGs were assessed by linear regression models, controlling for batch effect, age (years), sex, body mass index (BMI; < 25, 25.0 to < 30.0, ≥ 30.0 kg/m^2^), physical activity (inactive, insufficient, sufficient), and prevalence of CVD (ICD-10 codes I20–I16, I60–I69), diabetes (ICD-10 codes E10–E14), and cancer (ICD-10 codes C00–C99 except C44) at baseline. Dose–response relationships of current and lifetime smoking intensity, and time since smoking cessation with methylation intensity were assessed using restricted cubic spline (RSC) regression (Desquilbet and Mariotti 2010), controlling for the aforementioned confounders.

The associations of methylation intensities at each of the nine CpGs with all-cause mortality were first examined by Kaplan–Meier plots and log-rank tests. Then Cox regression models were fit adjusting for age (years), sex, and batch effect (model 1). Further models were additionally adjusted for smoking status (never, former, current smoker) (model 2) and for systolic blood pressure (millimeters of mercury), total cholesterol level (milligrams per deciliter), BMI (< 25, 25.0 to < 30.0, ≥ 30.0 kg/m^2^), physical activity (inactive, insufficient, sufficient), and prevalence of CVD (ICD-10 codes I20–I16, I60–I69), diabetes (ICD-10 codes E10–E14), and cancer (ICD-10 codes C00–C99 except C44) at baseline (model 3). Methylation intensity was entered into the models either as a categorical variable (using the highest quartiles as reference level) or as a continuous variable [calculating hazard ratios (HR) for a decrease in methylation intensity by one standard deviation]. In parallel, the associations between smoking at baseline and all-cause mortality were also estimated by Cox regression, with and without controlling for methylation intensities to explore the role of DNA methylation in smoking-related mortality. The proportional hazards assumption was assessed by martingale-based residuals (Lin et al. 1993). These preliminary analyses showed methylation at two of the nine CpGs (cg05575921, cg06126421) to be most strongly associated with all-cause mortality, whereas much less strong or nonsignificant associations were observed for the other seven CpGs. Additional preliminary analyses were conducted by *L_1_*-penalized Cox model (Benner et al. 2010; Goeman 2010) with nine CpGs and other risk factors as covariates; in that model, only cg05575921 and cg06126421 were selected among the nine CpGs. We therefore carried out analyses on all-cause and cause-specific mortality, including CVD, cancer, and other mortality, using a methylation-based score developed according to these two CpGs. Categories of the score were 2, 1, and 0 for participants in the lowest quartiles of both CpGs, in one of the two CpGs, and none of the two CpGs, respectively. In addition, the analyses were repeated after joint classification of participants according to both methylation score and sex.

To further assess the potential contributions of the smoking-associated CpGs for fatal cardiovascular risk prediction, methylation intensity at the nine CpGs individually and jointly added to a Cox regression model consisting of variables of the SCORE (Conroy et al. 2003), including age (years), sex, systolic blood pressure (millimeters of mercury), current smoking (yes, no), and total cholesterol (milligrams per deciliter) and using cardiovascular mortality as the dependent variable, additionally controlling for batch effect. Model fit was compared using Akaike information criterion (AIC) and likelihood ratio (LR) tests. Discrimination of the models was evaluated by Harrell’s *C*-statistics (Harrell et al. 1996), and the overoptimism was corrected using .632 bootstrap analysis with 1,000 replications [for this purpose, a SAS Macro was adapted from Miao’s work (Miao et al. 2013)]. Bootstrapping is a well-established approach for validation of a predictive model through quantifying the degradation in model predictive accuracy when applied in different data sources, which is known as overoptimism. The improvement in model performance by adding methylation intensity was examined by both net reclassification improvement (NRI) and integrated discrimination improvement (IDI). The NRI assesses whether participants are classified into clinically relevant risk categories by adding a new factor (e.g., methylation marker) to the risk prediction model (e.g., SCORE model). Absolute risk predictions were first calculated by Cox regression model with and without methylation marker for each individual, followed by assigning risk categories according to the recommended 10-year risk categories: 0–5%, > 5–10%, > 10–20%, and > 20% of predicted probability for a cardiovascular event (Cook 2007; Pencina et al. 2008). Movements are considered separately for cases (deaths) and controls (survivors), and deemed as correct direction if cases move into a higher risk category and controls move into a lower risk category. NRI = [(no. of cases up – no. of cases down)/no. of cases] – [(no. of controls up – no. of controls down)/no. of controls]. IDI estimates the mean difference in predicted probability for cases and controls over all possible cut-off points between models with and without methylation marker (Cook 2010; Pencina et al. 2008). Calibration of all assessed models was examined by May–Hosmer’s simplification of the Gronnesby–Borgan test (May and Hosmer 2004). The study population was divided into five subgroups according to the quintiles of the ranks based on their estimated risk probability, and model calibration was deemed satisfactory if *p*-values were > 0.05 for comparison of the observed and expected cases in each subgroup. Potential multicollinearity when simultaneously adding both CpGs in the model was assessed by variance inflation factor (VIF) and tolerance values, which did not indicate any relevant multicollinearity (e.g., VIF = 1.46 and tolerance = 0.69 when adding cg05575921 and cg06126421). Sensitivity analyses were carried out by excluding participants with prevalent CVD at baseline (*n* = 29).

The penalized Cox regression analyses were conducted using the R package “penalized” (version 0.9-42; Goeman et al. 2014), and all other analyses were carried out in SAS 9.3 (SAS Institute Inc., Cary, NC, USA).

## Results

Of 1,000 participants included in the present analysis, mortality follow-up was available for 999 subjects. Of the nine CpG sites assays, cg21566642, cg23576855, and cg21161138 had 3, 1, and 1 missing values, respectively; all other CpGs had complete data. Characteristics of the study population at baseline are shown in Table 1. Equal numbers of men and women of German nationality were included. The mean age was 62 years, and 33.9% of participants were younger than 60 years. More than half of the participants had ever smoked, and 19% still smoked at the time of recruitment, among whom male (61.3%) and younger (< 60 years, 45.2%) participants were somewhat overrepresented. During a median follow-up time of 10.3 years, 143 participants died. Among 135 participants with death certificates (94.4%), 50 died from CVD, 49 died from cancer, and 36 died from other diseases.

**Table 1:** Characteristics of the study population and methylation at *AHRR* (cg05575921) and *6p21.33* (cg06126421) (*n *= 1,000).*^a^*

Characteristic	*n* (%)	*AHRR *(cg05575921)			*6p21.33* (cg06126421)		
		Median	(Q1–Q3)	*p*-Value^*b*^	Median	(Q1–Q3)	*p*-Value^*b*^
Sex
Male	500 (50.0)	0.82	(0.70–0.87)		0.63	(0.57–0.69)
Female	500 (50.0)	0.88	(0.84–0.90)	< 0.0001	0.69	(0.65–0.73)	< 0.0001
Age (years)
< 60	339 (33.9)	0.85	(0.74–0.89)		0.61	(0.68–0.72)
60–64	289 (28.9)	0.86	(0.77–0.89)		0.66	(0.59–0.71)
65–69	226 (22.6)	0.86	(0.79–0.89)		0.66	(0.59–0.71)
70–75	146 (14.6)	0.86	(0.79–0.90)	0.20	0.66	(0.59–0.70)	0.20
Smoking status^*c*^
Never smoker	469 (48.0)	0.88	(0.86–0.90)		0.70	(0.66–0.73)
Former smoker	323 (33.0)	0.83	(0.77–0.87)		0.64	(0.58–0.69)
Current smoker	186 (19.0)	0.63	(0.56–0.70)	< 0.0001	0.57	(0.51–0.62)	< 0.0001
BMI (kg/m^2^)^*d*^
Underweight (< 18.5)	8 (0.8)	0.55	(0.66–0.87)		0.55	(0.50–0.66)
Normal weight (18.5 to < 25.0)	243 (24.4)	0.86	(0.73–0.89)		0.66	(0.59–0.71)
Overweight (25.0 to < 30.0)	483 (48.5)	0.86	(0.77–0.89)		0.67	(0.60–0.71)
Obese (≥ 30.0)	263 (26.4)	0.86	(0.78–0.89)	0.07	0.66	(0.60–0.72)	0.12
Physical activity^*e*^^,^^*f*^
Inactive	203 (20.3)	0.86	(0.74–0.89)		0.67	(0.59–0.71)
Insufficient	438 (43.8)	0.86	(0.77–0.89)		0.66	(0.59–0.71)
Sufficient	358 (35.8)	0.86	(0.78–0.89)	0.97	0.67	(0.60–0.72)	0.12
Diabetes^*e*^
Not prevalent	837 (83.8)	0.86	(0.77–0.89)		0.66	(0.59–0.71)
Prevalent	162 (16.2)	0.86	(0.78–0.89)	0.43	0.67	(0.60–0.72)	0.07
Cardiovascular disease^*e*^
Not prevalent	784 (78.4)	0.86	(0.78–0.89)		0.67	(0.60–0.71)
Prevalent	216 (21.6)	0.84	(0.74–0.88)	0.08	0.64	(0.58–0.69)	0.0003
Cancer
Not prevalent	934 (93.4)	0.86	(0.77–0.89)		0.66	(0.60–0.71)
Prevalent	66 (6.6)	0.86	(0.76–0.89)	0.71	0.65	(0.59–0.71)	0.37
Q, quartile. ^***a***^Data for the other seven CpGs are reported in Supplemental Material, Table S1. ^***b***^Kruskal–Wallis test for group differences. ^***c***^Data missing for 22 participants. ^***d***^Data missing for 3 participants. ^***e***^Data missing for 1 participant. ^***f***^Categories are defined as follows: inactive, < 1 hr/week of physical activity; sufficient: ≥ 2 hr/week of vigorous physical activity or ≥ 2 hr/week of light physical activity; insufficient, other.							

*Methylation intensities by demographic and behavioral factors*. Methylation intensities across various strata of characteristics of the study population are shown in Table 1 for *AHRR* cg05575921 and *6p21.33* cg06126421 (see Supplemental Material, Table S1, for all other CpGs). Men had lower methylation intensities than women at all nine CpG sites (all *p* < 0.0001). Methylation was not significantly associated with age (*p* > 0.05) except at *2q37.1* cg06644428 (*p* < 0.0001). Major differences were observed between never, former, and current smokers. Methylation levels at all nine CpGs were lower in current smokers than in never smokers and intermediate in former smokers, and all of the differences across the three group were statistically significant (*p* < 0.0001).

*Correlations of methylation intensities at the nine CpGs*. Mutual Spearman correlation coefficients for methylation intensities at all CpGs except cg06644428 were 0.46–0.93; Spearman correlation coefficients between cg06644428 and other CpGs were 0.18–0.66 (see Supplemental Material, Table S2).

**Figure 1:**
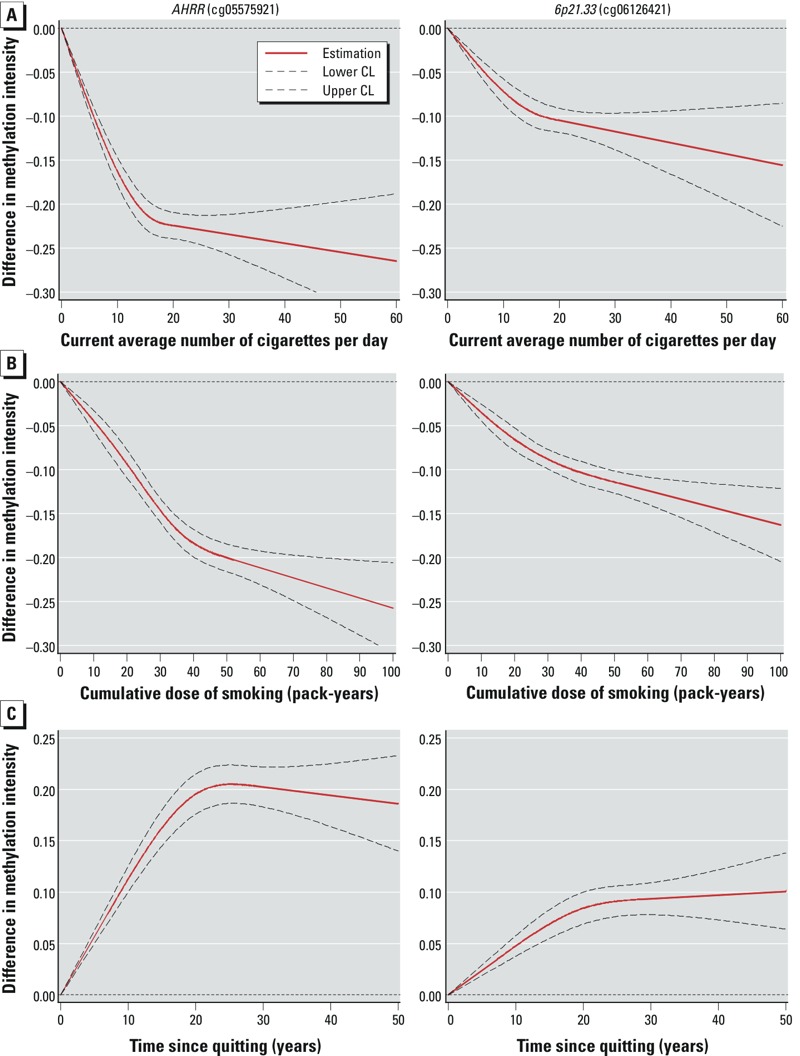
Dose–response relationships between smoking behavior and methylation intensity (results from restricted cubic spline regression adjusted for potential confounding factors). CL, confidence limit. (*A*) Dose–response relationship between current intensity of smoking and methylation intensity at *AHRR* (cg05575921; left), and *6p21.33 *(cg06126421; right); never and former smokers were defined as reference, with current smoking intensity = 0. (*B*) Dose–response relationship between cumulative dose of smoking and methylation intensity at *AHRR* (cg05575921; left), and *6p21.33 *(cg06126421; right); never smokers were defined as reference, with pack-years = 0. (*C*) Dose–response relationship between time since cessation of smoking and methylation intensity at *AHRR* (cg05575921; left), and *6p21.33 *(cg06126421; right) among former smokers; current smokers were defined as reference, with time since cessation = 0.

*Methylation intensities by smoking characteristics*. Table 2 shows the association between smoking behavior and methylation intensities at cg05575921 and cg06126421 estimated by linear regression (results for the other seven CpGs, which showed very similar patterns, are presented in Supplemental Material, Table S3). Compared with participants who never smoked, current and former smokers had the lowest and intermediate methylation levels at both CpGs, respectively. Methylation intensities were inversely associated with both current and lifetime smoking intensity, and were positively associated with time since cessation. Estimated dose–response curves for smoking behavior with methylation intensity at the two CpGs are shown in Figure 1. A steep decrease in methylation intensity was observed with increasing smoking intensity up to approximately 15 cigarettes per day and with increasing cumulative smoking up to approximately 30–40 pack-years, followed by further gradual decrease at higher current and lifetime smoking intensity. Among former smokers, methylation intensity steadily increased with time since cessation up to approximately 20–25 years after quitting and leveled off thereafter. Similar patterns of dose–response curves were also observed for most of the other seven CpGs [with exception of cg05951221, cg23576855, and cg06644428 for current smoking intensity; cg06644428 for pack-years; and cg23576855 and cg06644428 for time after quitting smoking (see Supplemental Material, Figure S1)].

**Table 2:** Association between smoking behavior and methylation intensity.*^a^*

Characteristic	*AHRR *(cg05575921)		*6p21.33* (cg06126421)	
	Regression coefficient	*p*‑Value	Regression coefficient	*p*‑Value
Smoking status^*b*^
Never smoker	Reference		Reference
Former smoker	–0.05 (–0.06, –0.04)	< 0.0001	–0.04 (–0.05, –0.03)	< 0.0001
Current smoker	–0.22 (–0.23, –0.20)	< 0.0001	–0.11 (–0.12, –0.10)	< 0.0001
Current intensity of smoking^*c*^ (average number of cigarettes/day)
0 (never and former smokers)	Reference		Reference
< 10	–0.14 (–0.17, –0.11)	< 0.0001	–0.06 (–0.09, –0.04)	< 0.0001
10–19	–0.20 (–0.22, –0.17)	< 0.0001	–0.08 (–0.10, –0.06)	< 0.0001
20–29	–0.22 (–0.23, –0.20)	< 0.0001	–0.11 (–0.12, –0.09)	< 0.0001
≥ 30	–0.27 (–0.31, –0.23)	< 0.0001	–0.13 (–0.17, –0.10)	< 0.0001
Cumulative dose of smoking (pack-years)^*d*^
0 (never smokers)	Reference		Reference
< 10	–0.03 (–0.05, –0.01)	0.001	–0.02 (–0.04, –0.01)	0.003
10–19	–0.09 (–0.10, –0.07)	< 0.0001	–0.06 (–0.07, –0.04)	< 0.0001
20–29	–0.12 (–0.13, –0.09)	< 0.0001	–0.08 (–0.09, –0.06)	< 0.0001
≥ 30	–0.19 (–0.21, –0.18)	< 0.0001	–0.11 (–0.12, –0.10)	< 0.0001
Time since cessation of smoking (years)^*e*^
0 (current smokers)	Reference		Reference
< 2	0.02 (–0.02, 0.06)	0.31	–0.002 (–0.04, 0.03)	0.93
2–4	0.11 (0.08, 0.13)	< 0.0001	0.04 (0.01, 0.06)	0.002
5–9	0.13 (0.11, 0.15)	< 0.0001	0.03 (0.01, 0.05)	0.007
10–20	0.17 (0.15, 0.19)	< 0.0001	0.07 (0.05, 0.08)	< 0.0001
≥ 20	0.21 (0.19, 0.22)	< 0.0001	0.09 (0.07, 0.10)	< 0.0001
^***a***^Results from linear regression, adjusted for sex, age, BMI (< 25 kg/m^2^, 25.0 to < 30.0 kg/m^2^, ≥ 30.0 kg/m^2^), physical activity ( inactive, insufficient, sufficient), prevalence of cardiovascular disease, diabetes, and cancer, and batch effect. ^***b***^Data missing for 22 participants. ^***c***^Data missing for 26 participants. ^***d***^Data missing for 68 participants. ^***e***^Data missing for 1 participant.				

*Methylation intensities and mortality*. Supplemental Material, Figure S2 depicts the survival experience according to quartiles of methylation intensity at the nine CpGs: a gradient of lower survival among participants with lower methylation levels was observed for 7 of the nine CpGs (all except cg23576855 and cg06644428). The associations of methylation intensity at the individual CpGs with all-cause mortality are further presented in Supplemental Material, Table S4. After multivariate adjustment, the strongest and statistically significant associations were estimated for two CpGs (cg05575921 and cg06126421), with HR = 2.45 [95% confidence interval (CI): 1.26, 4.79] and HR = 2.34 (95% CI: 1.27, 4.30), respectively, for the lowest quartile compared with the highest quartile. In addition, a decrease in methylation intensity by one standard deviation was associated with an increase in all-cause mortality by 15%–60% for seven CpGs (all except cg23576855 and cg06644428). In addition, a 1-SD decrease in methylation intensity was associated with higher all-cause mortality for seven CpGs (HR 1.15–1.59, with *p* < 0.05 for 5 CpGs); HRs for cg23576855 and cg06644428 were 0.97 and 1.00, respectively.

Table 3 shows the associations of score-based methylation with all-cause and cause-specific mortality. Multivariate-adjusted HRs for cardiovascular, cancer, and other mortality were 7.41 (95% CI: 2.81, 19.54), 2.48 (95% CI: 1.01, 6.08), and 2.78 (95% CI: 0.97, 7.98), respectively, for participants in the lowest quartile of methylation for both cg05575921 and cg06126421 compared with participants who were not in the lowest quartile of methylation for either CpG. By contrast, the strong associations between current smoking and all mortality outcomes were substantially attenuated or disappeared after adjustment for methylation-based score. Joint classification by sex and methylation demonstrated clear dose–response relationships of the methylation score with mortality in both sexes (see Supplemental Material, Table S5).

**Table 3:** Methylation score and smoking in relation to mortality outcomes.

Outcome/methylation score^*a*^/ smoking status	*n*_total_	Cases	PY	IR^*b*^	HR (95% CI)		
					Model 1^*c*^	Model 2^*d*^	Model 3^*e*^
All-cause mortality
0	677	60	6716.06	0.89	Reference	Reference	Reference
1	151	31	1431.15	2.17	2.08 (1.33, 3.25)	2.01 (1.25, 3.25)	1.90 (1.15, 3.14)
2	172	52	1546.03	3.36	3.41 (2.29, 5.08)	3.69 (2.21, 6.16)	3.59 (2.10, 6.16)
Never smoker	469	45	4651.73	0.97	Reference	Reference	Reference
Former smoker	323	58	3059.15	1.90	1.52 (1.00, 2.33)	1.14 (0.71, 1.80)	0.92 (0.56, 1.50)
Current smoker	186	37	1766.04	2.10	2.16 (1.37, 3.40)	0.91 (0.50, 1.63)	0.92 (0.50, 1.68)
CVD mortality
0	677	16	6690.70	0.24	Reference	Reference	Reference
1	151	14	1431.15	0.98	3.58 (1.69, 7.56)	4.37 (1.99, 9.61)	4.30 (1.89, 9.81)
2	172	20	1525.56	1.31	5.51 (2.68, 11.30)	9.25 (3.72, 22.96)	7.41 (2.81, 19.54)
Never smoker	469	17	4627.45	0.37	Reference	Reference	Reference
Former smoker	323	23	3058.06	0.75	1.50 (0.75, 3.00)	0.86 (0.40, 1.88)	0.70 (0.30, 1.64)
Current smoker	186	10	1745.58	0.57	1.59 (0.71, 3.58)	0.38 (0.14, 1.04)	0.44 (0.15, 1.24)
Cancer mortality
0	677	24	6690.70	0.36	Reference	Reference	Reference
1	151	9	1431.15	0.63	1.47 (0.67, 3.21)	1.15 (0.49, 2.70)	1.19 (0.48, 2.93)
2	172	16	1525.56	1.05	2.57 (1.31, 5.02)	2.06 (0.88, 4.79)	2.48 (1.01, 6.08)
Never smoker	469	14	4627.45	0.30	Reference	Reference	Reference
Former smoker	323	21	3058.06	0.69	1.86 (0.89, 3.89)	1.69 (0.77, 3.67)	1.37 (0.60, 3.01)
Current smoker	186	13	1745.58	0.74	2.43 (1.11, 5.35)	1.60 (0.59, 4.33)	1.45 (0.52, 4.08)
Other mortality
0	677	17	6690.70	0.22	Reference	Reference	Reference
1	151	6	1431.15	0.56	2.12 (0.88, 5.12)	1.88 (0.71, 4.95)	1.69 (0.62, 4.63)
2	172	11	1525.56	0.85	3.18 (1.45, 7.00)	2.86 (1.02, 8.04)	2.78 (0.97, 7.98)
Never smoker	469	15	4627.45	0.22	Reference	Reference	Reference
Former smoker	323	8	3058.06	0.43	1.46 (0.60, 3.57)	1.09 (0.42, 2.82)	0.83 (0.31, 2.23)
Current smoker	186	13	1745.58	0.63	2.81 (1.15, 6.89)	1.37 (0.43, 4.36)	1.35 (0.42, 4.38)
Abbreviations: HR, hazard ratio; IR, incidence rate; PY, person-years. ^***a***^Score was based on methylation intensity at cg05575921 and cg06126421, defined as follows: 2, methylation intensity in the lowest quartiles of both 2 CpG sites; 1, methylation intensity in the lowest quartiles of one of the 2 CpG sites; 0, other. ^***b***^Incidence rate per 100 person-years. ^***c***^Model 1: adjusted for age, sex, and batch effect. ^***d***^Model 2: model 1 plus adjusted for smoking status and methylation score. ^***e***^Model 3: model 2 plus adjusted for BMI, physical activity, systolic blood pressure, total cholesterol, hypertension, and prevalent CVD, diabetes, and cancer at baseline.							

*Methylation intensity and fatal cardiovascular risk prediction*. Table 4 and Supplemental Material, Table S6 present the increment in the performance indicators of the SCORE in prediction of fatal CVD by adding methylation intensity. The largest improvement was observed when including cg05575921 and cg06126421: Harrell’s *C*-statistics increased from 0.754 for the SCORE-only model to 0.822 and from 0.736 to 0.779 after correction for overoptimism (Table 4). Adding the two CpGs also resulted in 18 cases and 82 controls moving up and 11 cases and 151 controls moving down, which resulted in a NRI of 21.92% (*p* = 0.049) and a significant IDI of 3.73% (*p* = 0.005). Additionally adding methylation at other CpGs did not lead to a further improvement in the prediction of fatal CVD mortality (see Supplemental Material, Table S6). Even though NRI and IDI increased with additional CpGs included in the model, a substantial proportion of controls, who were supposed to move to lower risk categories, moved to higher risk categories along with cases moving to higher risk categories. The improvement in risk prediction became larger after excluding participants with CVD at baseline (*n* = 216; see Supplemental Material, Table S7). The Gronnesby–Borgan test indicated that the new model was also well-calibrated in both full and sensitivity analyses (all *p* > 0.05).

**Table 4:** Evaluation of the SCORE and methylation intensity in prediction of fatal CVD (controlling for batch effect).

Characteristic	SCORE	SCORE + cg05575921	SCORE + cg06126421	SCORE + cg05575921 + cg06126421
Overall model fit
–2 LOG L; df; *p*-value	623.93; 5; < 0.0001	601.82; 10; < 0.0001	602.21; 10; < 0.0001	597.54; 11; < 0.0001
AIC	633.93	621.82	622.52	619.54
LR test *p*-value^*a*^	—	0.0005	0.0006	0.0002
Harrell’s *C-*statistics (95% CI)	0.754 (0.691, 0.818)	0.810 (0.752, 0.867)	0.806 (0.748, 0.864)	0.822 (0.765, 0.879)
Optimism-corrected Harrell’s *C*-statistics (95% CI)	0.736 (0.676, 0.791)	0.773 (0.687, 0.832)	0.766 (0.678, 0.830)	0.779 (0.693, 0.840)
Reclassification of
Cases, *n*_up_/*n*_down_	Reference	18/11	18/12	18/11
Controls, *n*_up_/*n*_down_	Reference	86/157	88/146	82/151
NRI % (*p*-value)	Reference	22.14 (0.046)	18.66 (0.10)	21.92 (0.049)
IDI % (*p*-value)	Reference	3.39 (0.02)	3.36 (0.008)	3.73 (0.005)
Calibration
*n*_obs_/*n*_exp _(*p*-value)
Quintile 1	2/2 (0.82)	2/1 (0.40)	0/1 (0.26)	2/1 (0.40)
Quintile 2	3/4 (0.67)	2/3 (0.57)	3/3 (0.99)	1/3 (0.28)
Quintile 3	7/7 (0.94)	5/6 (0.82)	6/6 (0.73)	4/5 (0.74)
Quintile 4	10/12 (0.62)	7/10 (0.29)	8/10 (0.46)	7/10 (0.56)
Quintile 5	27/25 (0.68)	33/29 (0.45)	32/29 (0.55)	35/29 (0.30)
Abbreviations: AIC, Akaike information criterion; IDI, integrated discrimination improvement; LOG L, log-likelihood; LR, likelihood ratio; *n*_exp_, number of expected events; *n*_obs_, number of observed events; NRI, net reclassification improvement; SCORE, Systematic Coronary Risk Evaluation chart (age, sex, systolic blood pressure, current smoking, and total cholesterol). ^***a***^Comparison of SCORE + methylation model with SCORE model by likelihood ratio test.				

## Discussion

In this population-based cohort study, we found clear dose–response relationships of current and lifetime smoking exposure and time since smoking cessation with site-specific methylation, which were consistent among six CpGs located in *AHRR* (cg05575921, cg21161138), *F2RL3* (cg03636183), *2q37.1* (cg21566642, cg01940273), and *6p21.22* (cg06126421). Methylation at seven CpGs (all above + cg05951221) was also associated with mortality outcomes to various extents. A score based on methylation at the top two CpGs (cg05575921 and cg06126421) provided very strong associations with all-cause, cardiovascular, and cancer mortality. Moreover, integrating methylation at these two CpGs into the conventional risk factors substantially improved the accuracy of predicting fatal cardiovascular risk and reclassified a substantial proportion of individuals to higher or lower risk categories.

A biomarker reflecting long-term past smoking exposure is desirable for accurate evaluation of smoking cessation and for assessment of smoking-related disease risk (CDC 2010). DNA methylation biomarkers might be promising candidates for this purpose. Methylation at nine loci targeted in our study was reported to be strongly associated with smoking exposure by both previous genome-wide methylation studies (Shenker et al. 2013a; Zeilinger et al. 2013). In the present study, distinct and rather consistent dose–response patterns of methylation with respect to both lifetime cumulative smoking exposure and time since cessation were observed for six of the nine CpGs, which are, of note, similar to the dose–response patterns observed between smoking and smoking-related diseases. For example, cardiovascular risk increases sharply at low levels of cigarette consumption and then plateaus at higher levels of smoking (CDC 2010); the reduction of cardiovascular risk becomes evident within the initial years after quitting smoking and remains slightly elevated for more than a decade (CDC 2010; Kramer et al. 2006; Lightwood and Glantz 1997). The observed dose–response pattern of these six CpGs with current and lifetime smoking behavior was also consistent with dose–response patterns of methylation at the *F2RL3* gene previously identified by our group in a large study specifically focusing on this site (Zhang et al. 2014). In addition, in the study by Shenker et al. (2013b), a methylation index combining four of the nine CpGs investigated in our study (cg23576855, cg06644428, cg21566642, and cg06126421) provided superior performance in distinguishing former smokers from never smokers [area under the curve (AUC) = 0.82 (95% CI: 0.96, 0.99)] compared with cotinine [AUC = 0.47 (95% CI: 0.32, 0.63)]. Our present study, in which we addressed associations of methylation patterns with both smoking and smoking-related mortality, suggested that the identified DNA methylation biomarkers might be markers of cumulative smoking exposure-associated risk.

The *AHRR* gene, known as a tumor repressor (Zudaire et al. 2008), codes a protein involved in multiple pathophysiological pathways, such as metabolism of tobacco smoke components (Kasai et al. 2006; Moennikes et al. 2004) and regulation of cell proliferation and differentiation (Haarmann-Stemmann et al. 2007; Pot 2012). Hypomethylation of cg05575921 at *AHRR* has been reported to be associated with increasing lymphoblast *AHRR* gene expression *in vivo* (Monick et al. 2012). It has also been observed that *AHRR* expression in human lung tissues was inversly correlated with methylation levels of cg23576855 and cg21161138 at *AHRR*, with 5.7-fold increased expression in five current smokers compared with five nonsmokers (Shenker et al. 2013a). *AHRR* and the aryl hydrocarbon receptor (AHR) constitute a feedback loop in which the AHR heterodimer activates the expression of the *AHRR* gene, and the expressed *AHRR* inhibits the function of AHR in oncogenesis (Mimura et al. 1999). Tobacco smoking has been shown to trigger the production of AHR that mediates dioxin toxicity and other pathological effects (Martey et al. 2005; Meek and Finch 1999). Therefore, it is plausible to assume that demethylation/overexpression of the *AHRR* gene may result from a smoking-induced increase in AHR activation. The gene product of *F2RL3*, thrombin protease-activated receptor-4 (PAR-4), plays roles in inflammatory reactions and blood coagulation (Leger et al. 2006), and other pathophysiology commonly described in smoking-induced conditions (Leone 2007; Rahman and Laher 2007). Hypomethylation at *F2RL3* has been suggested to be strongly associated with mortality in a cohort of 1,206 patients with stable CVD (Breitling et al. 2012). Interestingly, methylation at four CpGs assessed in our study [*AHRR* (cg05575921), *F2RL3* (cg03636183), *2q37.1* (cg21566642), and *6p21.22* (cg06126421)] were recently found to be associated with a metabolic indicator of complex disorders, 4-vinylphenol sulfate (Petersen et al. 2014). Of note, this metabolic marker has also been reported to be associated with smoking (Manini et al. 2003). Although the potential joint or independent epigenetic role of the various loci remains to be clarified, these findings, as well as the disappearance or attenuation of association between smoking and mortality outcomes after adjustment for methylation at these CpGs in the present study, suggest that multiple DNA methylation sites are involved in mediating smoking-related adverse effects.

The much stronger associations of the methylation markers with mortality outcomes, compared with those of commonly studied molecular and genetic biomarkers, and the attenuation or disappearance of the association between current smoking and mortality after adjustment for the methylation markers observed in our study suggest that DNA methylation biomarkers may more accurately summarize individuals’ smoking-related risks that accumulated through past and current exposure, and thus be more informative in risk assessment than self-reported smoking history. To our knowledge, this is the first study to evaluate the improvement in risk assessment of fatal CVD when adding DNA methylation biomarkers to conventional risk factors. The increment in *C*-statistics by adding the methylation intensity at cg05575921 and cg06126421 (approximately 0.04) was much larger than the increment seen by adding a multimarker score in the Framingham Heart Study (*C*-statistics for model of major cardiovascular events increased by 0.01) (Wang et al. 2006). In another large population-based cohort, the investigators evaluated six novel biomarkers for cardiovascular risk prediction along with the conventional markers and reported the NRI was 0.00% and 4.70% for cardiovascular events and coronary events, respectively (Melander et al. 2009). They obtained improved NRI by restricting the analyses to individuals with intermediate risk; the reclassification, however, was essentially confined to down-classification of participants without events. Of note, the proportion of reclassified participants was substantial in our study, and consisted of not only down-classification of individuals without events but also up-classification of individuals with events. Given that nearly 22% of participants were reclassified, inclusion of smoking-associated methylation markers into the routine screening programs, such as the SCORE risk estimation system, would benefit a substantial proportion of individuals in the population setting and could greatly promote cost effectiveness of CVD prevention and therapy. On the other hand, our study was an exploratory investigation on CVD risk prediction using methylation markers based on a limited number of total cardiovascular deaths, thus our findings need to be validated in an independent population. The performance of these methylation markers for predicting risk of nonfatal or subtypes of fatal CVD, such as coronary and non-coronary heart disease, needs to be evaluated in further studies with high-quality assessment of CVD risk factors as well as CVD events. In addition, to examine the generalizability of the current finding, the performance of methylation markers should also be assessed in relation to other well-established risk scores, such as the Framingham score, and in geographically different populations.

Our study has specific strengths and limitations. Strengths of our study are the population-based prospective study design with comprehensive information on smoking exposure and a variety of covariates, as well as long-term complete mortality follow-up data. A limitation is that the limited numbers of cause-specific deaths prevent the analyses from going into more detail, such as sex-specific examination of CVD risk prediction or investigation of deaths from well-known smoking-associated subtypes of cancer (CDC 2014; Ezzati et al. 2005a). Future studies with large numbers of participants would be desirable to further validate our findings. Information on cause of death was based on death certificates, which are known to be less than perfect. However, potential misclassification between the broad categories of causes of deaths assessed in our study is likely to be much less relevant than potential misclassification between specific causes; given the rather consistent findings of an inverse association with methylation intensity for all categories of causes of deaths, such misclassification might have had only a small impact on the observed results. An additional limitation of our study is that methylation was measured from whole blood, without possibilities for differentiating DNA methylation between various cell types. It might therefore be conceivable that differences in methylation might, in part, reflect different distribution of leukocyte cell types. However, even if the difference in methylation we observed was primarily or partly due to shifts in leukocyte distribution, their use as biomarkers for characterizing smoking exposure or risk prediction would not be invalidated. On the contrary, given that DNA from whole blood is more readily obtainable in most clinical and epidemiological settings, biomarkers based on whole blood may be more relevant for clinical practice. Finally, our results are based on a single study and might be overoptimistic because only the CpG sites that performed best in the exploratory phase of the study were used to create the model and outcome classification. Further validation in independent studies should therefore be the aim for future studies.

Despite its limitations, our study strongly supports the potential utility of DNA methylation markers as indicators for both current and lifetime smoking exposure and for predicting mortality outcomes, in particular for cardiovascular mortality. Incorporation of methylation biomarkers into conventional risk factors might be a promising approach to improve cardiovascular risk assessment and disease prevention, which needs to be further validated and confirmed in additional studies with a large number of participants and detailed assessment of known determinants of CVD.

## Supplemental Material

(1.4 MB) PDF
