# Supplementary material for: Smoking-Associated DNA Methylation Biomarkers and Their Predictive Value for All-Cause and Cardiovascular Mortality
Source: Environ Health Perspect. 2015 May 27;124(1):67–74. doi: 10.1289/ehp.1409020 (PMC4710597; doi:10.1289/ehp.1409020)
Supplement: (1.4 MB) PDF [file ehp.1409020.s001.acco.pdf]

Environ Health Perspect
DOI: 10.1289/ehp.1409020

Note to Readers: *EHP* strives to ensure that all journal content is accessible to all readers. However, some figures and Supplemental Material published in *EHP* articles may not conform to 508 standards due to the complexity of the information being presented. If you need assistance accessing journal content, please contact ehp508@niehs.nih.gov. Our staff will work with you to assess and meet your accessibility needs within 3 working days.

Supplemental Material

Smoking-Associated DNA Methylation Biomarkers and Their Predictive Value for All-Cause and Cardiovascular Mortality

Yan Zhang, Ben Schöttker, Ines Florath, Christian Stock, Katja Butterbach, Bernd Holleczek, Ute Mons, and Hermann Brenner

Table of Contents

Table S1. Methylation intensity by characteristics of the study population
Table S2. Spearman correlation coefficients between methylation at 9 CpG sites
Table S3. Association between smoking behavior and methylation intensity
Table S4. Methylation intensity and smoking in relation to all-cause mortality
Table S5. Sex-specific association of the methylation score and smoking with all-cause mortality
Table S6. Evaluation of the SCORE and methylation intensity in prediction of fatal cardiovascular disease (controlling for batch effects)
Table S7. Evaluation of the SCORE and methylation intensity in prediction of fatal cardiovascular disease (excluding participants with cardiovascular disease at baseline n=216)
Figure S1. Dose-response relationships between smoking behavior and methylation intensity
Figure S2. Kaplan-Meier estimates of survival by methylation quartiles

Table S1. Methylation intensity by characteristics of the study population

Characteristics	*F2RL3* (cg03636183)			*AHRR* (cg23576855)			*AHRR* (cg21161138)		
	Median	(Q1 – Q3)	*P-value*[a]	Median	(Q1 – Q3)	*P-value*[a]	Median	(Q1 – Q3)	*P-value*[a]
Sex									
Male	0.67	(0.60 – 0.71)		0.64	(0.45 – 0.72)		0.70	(0.66 – 0.72)	
Female	0.70	(0.67 – 0.72)	<0.0001	0.73	(0.47 – 0.76)	<0.0001	0.72	(0.69 – 0.73)	<0.0001
Age (years)									
< 60	0.69	(0.62 – 0.71)		0.69	(0.45 – 0.75)		0.71	(0.67 – 0.73)	
60-64	0.69	(0.63 – 0.72)		0.69	(0.47 – 0.75)		0.71	(0.67 – 0.73)	
65-69	0.69	(0.64 – 0.72)		0.69	(0.44 – 0.75)		0.71	(0.67 – 0.73)	
70-75	0.68	(0.64 – 0.72)	0.75	0.71	(0.52 – 0.75)	0.60	0.71	(0.68 – 0.73)	0.50
Smoking status									
Never smokers	0.71	(0.68 – 0.73)		0.74	(0.69 – 0.76)		0.72	(0.70 – 0.74)	
Former smokers	0.67	(0.63 – 0.71)		0.66	(0.51 – 0.72)		0.70	(0.68 – 0.72)	
Current smokers	0.56	(0.52 – 0.63)	<0.0001	0.48	(0.39 – 0.55)	<0.0001	0.64	(0.62 – 0.68)	<0.0001
Body mass index (kg/m^2)									
Underweight (<18.5)	0.57	(0.53 – 0.69)		0.56	(0.44 – 0.73)		0.68	(0.60 – 0.71)	
Normal weight (18.5-<25.0)	0.68	(0.63 – 0.71)		0.70	(0.45 – 0.75)		0.71	(0.67 – 0.73)	
Overweight (25.0-<30.0)	0.69	(0.64 – 0.72)		0.69	(0.45 – 0.75)		0.71	(0.67 – 0.73)	
Obesity (≥30.0)	0.69	(0.64 – 0.71)	0.03	0.70	(0.49 – 0.75)	0.55	0.71	(0.68 – 0.73)	0.33
Physical activity[b]									
Inactive	0.69	(0.63 – 0.72)		0.66	(0.44 – 0.75)		0.71	(0.67 – 0.73)	
Insufficient	0.69	(0.63 – 0.72)		0.70	(0.47 – 0.75)		0.71	(0.67 – 0.73)	
Sufficient	0.69	(0.64 – 0.72)	0.52	0.69	(0.46 – 0.75)	0.28	0.71	(0.68 – 0.73)	0.83
Diabetes									
Not prevalent	0.69	(0.63 – 0.72)		0.70	(0.46 – 0.75)		0.71	(0.67 – 0.73)	
Prevalent	0.69	(0.64 – 0.72)	0.88	0.68	(0.44 – 0.74)	0.22	0.71	(0.67 – 0.73)	0.37
Cardiovascular disease									
Not prevalent	0.69	(0.64 – 0.72)		0.70	(0.45 – 0.75)		0.71	(0.67 – 0.73)	
Prevalent	0.68	(0.62 – 0.72)	0.47	0.68	(0.47 – 0.74)	0.47	0.70	(0.67 – 0.73)	0.11
Cancer									
Not prevalent	0.69	(0.64 – 0.72)		0.70	(0.46 – 0.75)		0.71	(0.67 – 0.73)	
Prevalent	0.67	(0.62 – 0.71)	0.13	0.69	(0.47 – 0.76)	0.92	0.71	(0.66 – 0.73)	0.63

Table S1. continued

Characteristics	*2q37.1* (cg06644428)			*2q37.1* (cg21566642)			*2q37.1* (cg05951221)			*2q37.1* (cg01940273)		
	Median	(Q1 – Q3)	*P-value*[a]	Median	(Q1 – Q3)	*P-value*[a]	Median	(Q1 – Q3)	*P-value*[a]	Median	(Q1 – Q3)	*P-value*[a]
Sex												
Male	0.10	(0.07 – 0.13)		0.43	(0.36 – 0.51)		0.38	(0.34 – 0.44)		0.58	(0.53 – 0.62)	
Female	0.12	(0.09 – 0.16)	<0.0001	0.51	(0.45 – 0.56)	<0.0001	0.44	(0.39 – 0.48)	<0.0001	0.62	(0.58 – 0.65)	<0.0001
Age (years)												
< 60	0.10	(0.07 – 0.13)		0.48	(0.37 – 0.54)		0.41	(0.35 – 0.46)		0.60	(0.55 – 0.64)	
60-64	0.11	(0.08 – 0.15)		0.48	(0.41 – 0.54)		0.42	(0.36 – 0.47)		0.60	(0.56 – 0.64)	
65-69	0.12	(0.08 – 0.16)		0.48	(0.41 – 0.55)		0.41	(0.36 – 0.46)		0.60	(0.56 – 0.63)	
70-75	0.12	(0.09 – 0.17)	<0.0001	0.48	(0.40 – 0.54)	0.50	0.42	(0.37 – 0.47)	0.13	0.60	(0.56 – 0.63)	0.94
Smoking status												
Never smokers	0.13	(0.10 – 0.17)		0.53	(0.48 – 0.57)		0.46	(0.42 – 0.49)		0.63	(0.60 – 0.65)	
Former smokers	0.09	(0.07 – 0.13)		0.44	(0.39– 0.50)		0.38	(0.35 – 0.44)		0.59	(0.55 – 0.62)	
Current smokers	0.08	(0.06 – 0.11)	<0.0001	0.34	(0.30 – 0.39)	<0.0001	0.33	(0.30 – 0.47)	<0.0001	0.51	(0.48 – 0.55)	<0.0001
Body mass index (kg/m^2)												
Underweight (<18.5)	0.08	(0.05 – 0.14)		0.41	(0.30 – 0.50)		0.35	(0.29 – 0.44)		0.53	(0.47 – 0.59)	
Normal weight (18.5-<25.0)	0.11	(0.09 – 0.15)		0.48	(0.38 – 0.56)		0.42	(0.35 – 0.47)		0.60	(0.54 – 0.64)	
Overweight (25.0-<30.0)	0.11	(0.08 – 0.15)		0.48	(0.40 – 0.54)		0.42	(0.36 – 0.46)		0.60	(0.56 – 0.63)	
Obesity (≥30.0)	0.11	(0.08 – 0.17)	0.33	0.48	(0.40 – 0.54)	0.53	0.41	(0.36 – 0.46)	0.31	0.60	(0.56 – 0.64)	0.14
Physical activity[b]												
Inactive	0.11	(0.08 – 0.15)		0.48	(0.38 – 0.54)		0.41	(0.35 – 0.47)		0.60	(0.55 – 0.64)	
Insufficient	0.11	(0.08 – 0.14)		0.47	(0.39 – 0.54)		0.41	(0.35 – 0.46)		0.60	(0.55 – 0.64)	
Sufficient	0.11	(0.08 – 0.15)	0.47	0.48	(0.42 – 0.55)	0.21	0.42	(0.37 – 0.46)	0.37	0.60	(0.56 – 0.64)	0.73
Diabetes												
Not prevalent	0.11	(0.08 – 0.15)		0.48	(0.40 – 0.55)		0.42	(0.36 – 0.46)		0.60	(0.55 – 0.64)	
Prevalent	0.10	(0.07 – 0.14)	0.20	0.46	(0.39 – 0.53)	0.24	0.40	(0.36 – 0.46)	0.31	0.60	(0.56 – 0.63)	0.80
Cardiovascular disease												
Not prevalent	0.11	(0.08 – 0.15)		0.48	(0.40 – 0.55)		0.42	(0.36 – 0.46)		0.60	(0.56 – 0.64)	
Prevalent	0.11	(0.08 – 0.16)	0.72	0.46	(0.38 – 0.53)	0.09	0.40	(0.35 – 0.47)	0.19	0.59	(0.55 – 0.63)	0.11
Cancer												
Not prevalent	0.11	(0.08 – 0.15)		0.48	(0.40 – 0.54)		0.42	(0.36 – 0.46)		0.60	(0.55 – 0.64)	
Prevalent	0.12	(0.09 – 0.15)	0.14	0.48	(0.38 – 0.53)	0.55	0.42	(0.36 – 0.46)	0.83	0.60	(0.56 – 0.63)	0.94

[a]Kruskal–Wallis test for group differences. [b]categories defined as follows: inactive, < 1 hr/week of physical activity; medium/high: ≥ 2 hr/week of vigorous physical activity or ≥ 2 hr/week of light physical activity; low, other.

Table S2. Spearman correlation coefficients between methylation at 9 CpG sites

CpG sites	Spearman correlation coefficients<br>P-value<br>N								
	F2RL3 **cg03636183**	***AHRR*** **cg23576855**	***AHRR*** **cg21161138**	***AHRR*** **cg05575921**	***2q37.1*** **cg06644428**	***2q37.1*** **cg21566642**	***2q37.1*** **cg05951221**	***2q37.1*** **cg01940273**	***6p21.33*** **cg06126421**
F2RL3	1.00000								
**cg03636183**									
	1000								
AHRR	0.51275	1.00000							
**cg23576855**	<.0001								
	999	999							
AHRR	0.58539	0.45553	1.00000						
**cg21161138**	<.0001	<.0001							
	999	998	999						
AHRR	0.69655	0.49972	0.67018	1.00000					
**cg05575921**	<.0001	<.0001	<.0001						
	1000	999	999	1000					
2q37.1	0.39970	0.30266	0.17978	0.38411	1.00000				
**cg06644428**	<.0001	<.0001	<.0001	<.0001					
	1000	999	999	1000	1000				
2q37.1	0.62415	0.51388	0.56967	0.71401	0.65206	1.00000			
**cg21566642**	<.0001	<.0001	<.0001	<.0001	<.0001				
	997	996	996	997	997	997			
2q37.1	0.62257	0.51224	0.54598	0.68458	0.71755	0.93306	1.00000		
**cg05951221**	<.0001	<.0001	<.0001	<.0001	<.0001	<.0001			
	1000	999	999	1000	1000	997	1000		
2q37.1	0.65822	0.53661	0.59022	0.67386	0.54746	0.90002	0.87641	1.00000	
**cg01940273**	<.0001	<.0001	<.0001	<.0001	<.0001	<.0001	<.0001		
	1000	999	999	1000	1000	997	1000	1000	
6p21.33	0.65740	0.45793	0.46465	0.61236	0.37286	0.56921	0.55514	0.60290	1.00000
**cg06126421**	<.0001	<.0001	<.0001	<.0001	<.0001	<.0001	<.0001	<.0001	
	1000	999	999	1000	1000	997	1000	1000	1000

Table S3. Association between smoking behavior and methylation intensity[a]

Smoking characteristic	*F2RL3* (cg03636183)		*AHRR* (cg23576855)		*AHRR* (cg21161138)	
	Regression coefficient	*P-value*	Regression coefficient	*P-value*	Regression coefficient	*P-value*
Smoking status						
Never smoker	Ref.		Ref.		Ref.	
Former smoker	-0.03 (-0.04, -0.02)	<0.0001	-0.05 (-0.07, -0.03)	< 0.0001	-0.01 (-0.02, -0.009)	<0.0001
Current smoker	-0.12 (-0.13, -0.11)	<0.0001	-0.19 (-0.22, -0.17)	< 0.0001	-0.07 (-0.08, -0.06)	<0.0001
Current intensity of smoking (average number of cigarettes /day)						
0 (Never and formersmokers)	Ref.		Ref.		Ref.	
<10	-0.08 (-0.10, -0.06)	<0.0001	-0.12 (-0.17, -0.07)	<0.0001	-0.05 (-0.06, -0.03)	<0.0001
10-19	-0.11 (-0.13, -0.09)	<0.0001	-0.19 (-0.24, -0.14)	<0.0001	-0.06 (-0.08, -0.05)	<0.0001
20-29	-0.12 (-0.14, -0.11)	<0.0001	-0.20 (-0.23, -0.16)	<0.0001	-0.07 (-0.08, -0.06)	<0.0001
≥30	-0.16 (-0.18, -0.13)	<0.0001	-0.19 (-0.26, -0.12)	<0.0001	-0.09 (-0.11, -0.07)	<0.0001
Cumulative dose of smoking (pack-years)						
0 (Never smokers)	Ref.		Ref.		Ref.	
<10	-0.02 (-0.03, -0.01)	0.002	-0.07 (-0.11, -0.03)	0.0002	-0.01 (-0.02, -0.003)	0.01
10-19	-0.05 (-0.07, -0.04)	<0.0001	-0.07 (-0.11, -0.04)	<0.0001	-0.03 (-0.04, -0.02)	<0.0001
20-29	-0.07 (-0.08, -0.05)	<0.0001	-0.10 (-0.13, -0.07)	<0.0001	-0.03 (-0.04, -0.02)	<0.0001
≥30	-0.12 (-0.13,-0.11)	<0.0001	-0.16 (-0.18, -0.13)	<0.0001	-0.06 (-0.07, -0.05)	<0.0001
Time since cessation of smoking (years)						
0 (Current smokers)	Ref.		Ref.		Ref.	
<2	-0.005 (-0.03, 0.02)	0.70	0.05 (-0.02, 0.13)	0.21	0.006 (-0.01, 0.03)	0.57
2-4	0.06 (0.04, 0.08)	<0.0001	0.13 (0.07, 0.18)	<0.0001	0.04 (0.03, 0.05)	<0.0001
5-9	0.05 (0.04, 0.07)	<0.0001	0.13 (0.08, 0.18)	<0.0001	0.03 (0.02, 0.04)	<0.0001
10-20	0.09 (0.08, 0.10)	<0.0001	0.16 (0.12, 0.20)	<0.0001	0.06 (0.05, 0.07)	<0.0001
≥20	0.12 (0.10, 0.13)	<0.0001	0.15 (0.11, 0.18)	<0.0001	0.06 (0.05, 0.07)	<0.0001

Table S3. continued

Smoking characteristic	*2q37.1* (cg06644428) Regression coefficient	*P-value*	*2q37.1* (cg21566642) Regression coefficient	*P-value*
Smoking status				
Never smoker	Ref.		Ref.	
Former smoker	-0.03 (-0.04, -0.02)	<0.0001	-0.06 (-0.07, -0.05)	< 0.0001
Current smoker	-0.04 (-0.05, -0.03)	<0.0001	-0.17 (-0.18, -0.15)	< 0.0001
Current intensity of smoking (average number of cigarettes /day)				
0 (Never and former smokers)	Ref.		Ref.	
<10	-0.03 (-0.04, -0.01)	0.002	-0.10 (-0.13, -0.08)	<0.0001
10-19	-0.03 (-0.04, -0.01)	0.0002	-0.14 (-0.17, -0.12)	<0.0001
20-29	-0.03 (-0.04, -0.02)	<0.0001	-0.16 (-0.17, -0.14)	<0.0001
≥30	-0.03 (-0.05, -0.008)	0.008	-0.17 (-0.21, -0.14)	<0.0001
Cumulative dose of smoking (pack-years)				
0 (Never smokers)	Ref.		Ref.	
<10	-0.02 (-0.03, -0.004)	0.006	-0.04 (-0.06, -0.02)	<0.0001
10-19	-0.04 (-0.05, -0.03)	<0.0001	-0.09 (-0.10, -0.07)	<0.0001
20-29	-0.03 (-0.04, -0.02)	<0.0001	-0.11 (-0.13, -0.09)	<0.0001
≥30	-0.04 (-0.05, -0.03)	<0.0001	-0.16 (-0.17, -0.14)	<0.0001
Time since cessation of smoking (years)				
0 (Current smokers)	Ref.		Ref.	
<2	-0.01 (-0.04, 0.01)	0.30	0.01 (-0.03, 0.05)	0.62
2-4	0.02 (0.01, 0.04)	0.008	0.08 (0.05, 0.11)	<0.0001
5-9	0.004 (-0.01, 0.02)	0.59	0.06 (0.04, 0.08)	<0.0001
10-20	0.01 (-0.001, 0.02)	0.07	0.10 (0.09, 0.12)	<0.0001
≥20	0.02 (0.01, 0.03)	<0.0001	0.13 (0.12, 0.15)	<0.0001

[a]Results from linear regression, adjusted for sex, age, BMI (<25 kg/m2 / 25.0-<30.0 kg/m2 / ≥30.0 kg/m2), physical activity (inactive/low/medium and high), prevalence of cardiovascular disease, diabetes, and cancer, and batch effect.

Table S3. continued

Smoking characteristic	*2q37.1* (cg05951221) Regression coefficient	*P-value*	*2q37.1* (cg01940273) Regression coefficient	*P-value*
Smoking status				
Never smoker	Ref.		Ref.	
Former smoker	-0.05 (-0.06, -0.04)	<0.0001	-0.03 (-0.04, -0.02)	<0.0001
Current smoker	-0.11 (-0.12, -0.10)	<0.0001	-0.10 (-0.11, -0.09)	<0.0001
Current intensity of smoking (average number of cigarettes /day)				
0 (Never and former smokers)	Ref.		Ref.	
<10	-0.07 (-0.09, -0.05)	<0.0001	-0.07 (-0.09, -0.05)	<0.0001
10-19	-0.08 (-0.10, -0.06)	<0.0001	-0.08 (-0.09, -0.07)	<0.0001
20-29	-0.09 (-0.11, -0.08)	<0.0001	-0.10 (-0.11, -0.08)	<0.0001
≥30	-0.09 (-0.12, -0.07)	<0.0001	-0.11 (-0.13, -0.09)	<0.0001
Cumulative dose of smoking (pack-years)				
0 (Never smokers)	Ref.		Ref.	
<10	-0.03 (-0.04, -0.02)	<0.0001	-0.02 (-0.04, -0.01)	<0.0001
10-19	-0.06 (-0.07, -0.05)	<0.0001	-0.04 (-0.06, -0.03)	<0.0001
20-29	-0.07 (-0.09, -0.06)	<0.0001	-0.06 (-0.07, -0.04)	<0.0001
≥30	-0.11 (-0.12, -0.10)	<0.0001	-0.09 (-0.10, -0.08)	<0.0001
Time since cessation of smoking (years)				
0 (Current smokers)	Ref.		Ref.	
<2	0.004 (-0.02, 0.03)	0.77	0.02 (-0.005, 0.04)	0.12
2-4	0.04 (0.02, 0.06)	<0.0001	0.05 (0.03, 0.07)	<0.0001
5-9	0.03 (0.01, 0.05)	0.001	0.05 (0.03, 0.06)	<0.0001
10-20	0.05 (0.04, 0.07)	<0.0001	0.07 (0.06, 0.08)	<0.0001
≥20	0.08 (0.07, 0.09)	<0.0001	0.09 (0.08, 0.10)	<0.0001

[a]Results from linear regression, adjusted for sex, age, BMI (<25 kg/m2 / 25.0-<30.0 kg/m2 / ≥30.0 kg/m2), physical activity (inactive/low/medium and high), prevalence of cardiovascular disease, diabetes, and cancer, and batch effect.

Table S4. Methylation intensity and smoking in relation to all-cause mortality

CpG sites	Methylation level and Smoking status	HR (95% CI)		
		Model 1[a]	Model 2[b]	Model 3[c]
F2RL3 (cg03636183)	>0.72 (Quartile 4)	Ref.	Ref.	Ref.
	≤0.72 (Quartile 3)	1.48 (0.82, 2.66)	1.43 (0.79, 2.59)	1.31 (0.72, 2.39)
	≤0.69 (Quartile 2)	1.49 (0.83, 2.68)	1.32 (0.72, 2.41)	1.23 (0.67, 2.26)
	≤0.64 (Quartile 1)	2.73 (1.58, 4.72)	2.29 (1.22, 4.30)	1.55 (0.81, 2.96)
	Per STD less methylation	1.42 (1.23, 1.64)	1.40 (1.16, 1.69)	1.30 (1.06, 1.60)
	Never smoker	Ref.	Ref.	Ref.
	Former smoker	1.52 (1.00, 2.33)	1.25 (0.79, 1.98)	1.16 (0.72, 1.86)
	Current smoker	2.16 (1.37, 3.40)	1.37 (0.77, 2.44)	1.73 (0.97, 3.10)
AHRR (cg23576855)	>0.75 (Quartile 4)	Ref.	Ref.	Ref.
	≤0.75 (Quartile 3)	1.07 (0.62, 1.86)	0.95 (0.54, 1.67)	0.86 (0.49, 1.53)
	≤0.69 (Quartile 2)	1.96 (1.15, 3.32)	1.34 (0.74, 2.43)	1.12 (0.61, 2.08)
	≤0.45 (Quartile 1)	1.21 (0.69, 2.11)	0.94 (0.51, 1.70)	0.75 (0.40, 1.41)
	Per STD less methylation	1.14 (0.97, 1.34)	1.02 (0.84, 1.23)	0.97 (0.79, 1.19)
	Never smoker	Ref.	Ref.	Ref.
	Former smoker	1.52 (1.00, 2.33)	1.44 (0.91, 2.28)	1.24 (0.77, 1.99)
	Current smoker	2.16 (1.37, 3.40)	1.92 (1.13, 3.29)	2.10 (1.21, 3.65)
AHRR (cg21161138)	>0.73 (Quartile 4)	Ref.	Ref.	Ref.
	≤0.73 (Quartile 3)	0.85 (0.49, 1.48)	0.82 (0.47, 1.44)	0.65 (0.36, 1.16)
	≤0.71 (Quartile 2)	1.10 (0.66, 1.82)	1.00 (0.59, 1.68)	0.85 (0.50, 1.45)
	≤0.67 (Quartile 1)	1.99 (1.23, 3.23)	1.62 (0.92, 2.85)	1.22 (0.68, 2.18)
	Per STD less methylation	1.44 (1.25, 1.66)	1.38 (1.16, 1.63)	1.29 (1.08, 1.55)
	Never smoker	Ref.	Ref.	Ref.
	Former smoker	1.52 (1.00, 2.33)	1.46 (0.94, 2.28)	1.24 (0.79, 1.96)
	Current smoker	2.16 (1.37, 3.40)	1.56 (0.90, 2.70)	1.73 (1.00, 2.99)
AHRR (cg05575921)	>0.89 (Quartile 4)	Ref.	Ref.	Ref.
	≤0.89 (Quartile 3)	0.70 (0.37, 1.33)	0.72 (0.38, 1.37)	0.68 (0.35, 1.30)
	≤0.86 (Quartile 2)	1.29 (0.75, 2.23)	1.30 (0.73, 2.30)	1.02 (0.56, 1.84)
	≤0.77 (Quartile 1)	2.80 (1.67, 4.69)	3.09 (1.63, 5.85)	2.45 (1.26, 4.79)
	Per STD less methylation	1.53 (1.33, 1.77)	1.69 (1.37, 2.09)	1.59 (1.27, 1.99)
	Never smoker	Ref.	Ref.	Ref.
	Former smoker	1.52 (1.00, 2.33)	1.09 (0.67, 1.76)	0.96 (0.58, 1.56)
	Current smoker	2.16 (1.37, 3.40)	0.87 (0.47, 1.59)	0.98 (0.53, 1.81)
2q37.1 (cg06644428)	>0.15 (Quartile 4)	Ref.	Ref.	Ref.
	≤0.15 (Quartile 3)	1.00 (0.60, 1.67)	0.98 (0.59, 1.66)	0.98 (0.58, 1.66)
	≤0.11 (Quartile 2)	1.14 (0.68, 1.92)	1.01 (0.59, 1.72)	1.01 (0.58, 1.76)
	≤0.08 (Quartile 1)	1.61 (1.00, 2.60)	1.30 (0.78, 2.17)	1.23 (0.72, 2.11)
	Per STD less methylation	1.08 (0.91, 1.29)	1.02 (0.85, 1.23)	1.00 (0.83, 1.20)
	Never smoker	Ref.	Ref.	Ref.
	Former smoker	1.52 (1.00, 2.33)	1.47 (0.94, 2.29)	1.22 (0.77, 1.93)
	Current smoker	2.16 (1.37, 3.40)	2.03 (1.25, 3.29)	2.00 (1.22, 3.28)

Table S4. continued

CpG sites	Methylation level and Smoking status	HR (95% CI)		
		Model 1[a]	Model 2[b]	Model 3[c]
2q37.1 (cg21566642)	>0.54 (Quartile 4)	Ref.	Ref.	Ref.
	≤0.54 (Quartile 3)	1.18 (0.66, 2.13)	1.12 (0.62, 2.03)	0.93 (0.50, 1.71)
	≤0.48 (Quartile 2)	1.54 (0.90, 2.63)	1.34 (0.76, 2.34)	1.20 (0.67, 2.14)
	≤0.40 (Quartile 1)	2.53 (1.51, 4.24)	2.12 (1.16, 3.87)	1.67 (0.90, 3.10)
	Per STD less methylation	1.43 (1.19, 1.71)	1.31 (1.03, 1.66)	1.20 (0.94, 1.53)
	Never smoker	Ref.	Ref.	Ref.
	Former smoker	1.52 (1.00, 2.33)	1.23 (0.78, 1.97)	1.04 (0.64, 1.68)
	Current smoker	2.16 (1.37, 3.40)	1.36 (0.77, 2.40)	1.46 (0.82, 2.59)
2q37.1 (cg05951221)	>0.46 (Quartile 4)	Ref.	Ref.	Ref.
	≤0.46 (Quartile 3)	0.71 (0.38, 1.31)	0.59 (0.31, 1.13)	0.51 (0.26, 1.01)
	≤0.42 (Quartile 2)	1.27 (0.78, 2.07)	1.10 (0.66, 1.84)	0.94 (0.55, 1.61)
	≤0.36 (Quartile 1)	1.83 (1.13, 2.95)	1.36 (0.78, 2.37)	1.33 (0.75, 2.35)
	Per STD less methylation	1.35 (1.12, 1.62)	1.20 (0.96, 1.49)	1.15 (0.91, 1.44)
	Never smoker	Ref.	Ref.	Ref.
	Former smoker	1.52 (1.00, 2.33)	1.34 (0.84, 2.12)	1.10 (0.68, 1.77)
	Current smoker	2.16 (1.37, 3.40)	1.65 (0.96, 2.85)	1.60 (0.92, 2.79)
2q37.1 (cg01940273)	>0.64 (Quartile 4)	Ref.	Ref.	Ref.
	≤0.64 (Quartile 3)	1.20 (0.66, 2.17)	1.07 (0.58, 1.95)	1.03 (0.56, 1.90)
	≤0.60 (Quartile 2)	1.73 (0.98, 3.07)	1.49 (0.82, 2.70)	1.31 (0.71, 2.41)
	≤0.55 (Quartile 1)	2.40 (1.36, 4.22)	1.88 (0.99, 3.58)	1.65 (0.85, 3.20)
	Per STD less methylation	1.44 (1.21, 1.72)	1.37 (1.09, 1.72)	1.29 (1.02, 1.63)
	Never smoker	Ref.	Ref.	Ref.
	Former smoker	1.52 (1.00, 2.33)	1.35 (0.86, 2.11)	1.14 (0.71, 1.81)
	Current smoker	2.16 (1.37, 3.40)	1.53 (0.88, 2.67)	1.58 (0.90, 2.79)
6p21.33 (cg06126421)	>0.71 (Quartile 4)	Ref.	Ref.	Ref.
	≤0.71 (Quartile 3)	1.09 (0.59, 2.00)	1.02 (0.55, 1.90)	1.00 (0.53, 1.88)
	≤0.66 (Quartile 2)	1.54 (0.86, 2.78)	1.38 (0.75, 2.54)	1.20 (0.65, 2.24)
	≤0.60 (Quartile 1)	3.09 (1.80, 5.31)	2.67 (1.48, 4.82)	2.34 (1.27, 4.30)
	Per STD less methylation	1.57 (1.34, 1.85)	1.51 (1.25, 1.83)	1.45 (1.19, 1.78)
	Never smoker	Ref.	Ref.	Ref.
	Former smoker	1.52 (1.00, 2.33)	1.26 (0.80, 1.96)	1.04 (0.65, 1.67)
	Current smoker	2.16 (1.37, 3.40)	1.33 (0.79, 2.22)	1.31 (0.76, 2.25)

Abbreviation: CI, confidence interval; HR, hazard ratio; Ref., reference category; STD, standard deviation.
[a]Model1: adjusted for age, sex and batch effect; [b]Model 2: like model 1, additionally adjusted for smoking status/methylation intensity (quartiles); [c]Model 3: like model 2, additionally adjusted for body mass index, physical activity, systolic blood pressure, total cholesterol, hypertension, and prevalent cardiovascular disease, diabetes, and cancer at baseline.

Table S5. Sex-specific association of the methylation score and smoking with all-cause mortality

Sex + Methylation score[a]/ Smoking status	N_{total}	Cases	PY	IR[b]	HR (95% CI)		
					Model 1[c]	Model 2[d]	Model 3[e]
Female with score = 0	408	28	4124.90	0.68	Ref.	Ref.	Ref.
Female with score = 1	52	9	512.53	1.76	2.76 (1.30, 5.86)	2.81 (1.28, 6.14)	2.76 (1.24, 6.15)
Female with score = 2	39	8	376.22	2.13	3.90 (1.77, 8.57)	4.19 (1.68, 10.49)	3.76 (1.75, 9.60)
Male with score = 0	268	32	2591.17	1.23	2.01 (1.21, 3.35)	1.82 (1.06, 3.14)	1.96 (1.11, 3.45)
Male with score = 1	99	22	918.62	2.39	3.64 (2.08, 6.37)	3.17 (1.69, 5.95)	3.26 (1.69, 6.29)
Male with score = 2	133	44	1169.81	3.76	6.07 (3.77, 9.75)	6.10 (3.28, 11.33)	6.40 (3.36, 12.21)
Never smoker	469	45	4651.73	0.97	Ref.	Ref.	Ref.
Former smoker	323	58	3059.15	1.90	1.52 (1.00, 2.33)	1.07 (0.67,1.70)	0.88 (0.54, 1.43)
Current smoker	186	37	1766.04	2.10	2.16 (1.37, 3.40)	0.86 (0.47, 1.56)	0.89 (0.49, 1.64)

Abbreviations: HR, hazard ratio; CI, confidence interval; IR, incidence rate; PY, person-years; Ref., reference category. [a]Score was based on methylation intensity at cg05575921 and cg06126421, defined as follows: 2, methylation intensity in the lowest quartiles of both 2 CpG sites; 1, methylation intensity in the lowest quartiles of one of the 2 CpG sites; 0, other. [b]Incidence rate per 100 person-years. [c]Model1: adjusted for age; [d]Model 2: like model 1, additionally adjusted for smoking status/methylation score+sex; [e]Model 3: like model 2, additionally adjusted for BMI, physical activity, systolic blood pressure, total cholesterol, hypertension, and prevalent cardiovascular disease, diabetes, and cancer at baseline.

Table S6. Evaluation of the SCORE and methylation intensity in prediction of fatal cardiovascular disease (controlling for batch effects)

	Overall model fit			Harrell's	Reclassification				Calibration n_{obs}/n_{exp}(p-value)				
	-2 LOG L;df; p-value	AIC	LR test p-value[a]	C statistics (95% CI)	Cases n_{up}/n_{down}	Controls n_{up}/n_{down}	NRI % (p-value)	IDI % (p-value)	Quintile1	Quintile 2	Quintile 3	Quintile 4	Quintile 5
SCORE	623.93; 5; <.0001	633.93	---	0.754(0.691, 0.818)<br>0.736 (0.676, 0.791)[b]	Ref.	Ref.	Ref.	Ref.	2/2 (0.82)	3/4 (0.67)	7/7 (0.94)	10/12 (0.62)	27/25 (0.68)
SCORE + cg03636183	609.53; 10; <.0001	629.53	0.01	0.799 (0.743, 0.856)<br>0.759 (0.674, 0.818)[b]	13/10	81/137	12.32 (0.21)	1.73 (0.06)	1/1 (0.77)	3/3 (0.90)	3/6 (0.24)	13/11 (0.49)	29/28 (0.83)
SCORE + cg21566642	612.69; 10; <.0001	632.69	0.05	0.792 (0.732, 0.851)<br>0.749 (0.668, 0.812)[b]	13/9	74/130	14.38 (0.14)	1.00 (0.17)	2/1 (0.59)	3/3 (0.86)	3/6 (0.22)	12/11 (0.76)	19/27 (0.75)
SCORE + cg05951221	608.52; 10; <.0001	628.52	0.009	0.796 (0.736, 0.856)<br>0.757 (0.671, 0.818)[b]	14/7	82/140	20.70 (0.03)	2.02 (0.04)	3/1 (0.11)	3/3 (0.98)	1/6 (0.05)	11/11 (1.00)	31/28 (0.60)
SCORE + cg23576855	617.77; 10; <.0001	637.77	0.29	0.770 (0.708, 0.833)<br>0.728 (0.647, 0.791)[b]	5/8	59/95	-2.14 (0.78)	0.84 (0.19)	3/2 (0.21)	2/3 (0.44)	6/6 (0.93)	10/12 (0.65)	28/26 (0.74)
SCORE + cg21161138	616.90; 10; <.0001	636.90	0.21	0.769 (0.708, 0.831)<br>0.728 (0.646, 0.792)[b]	9/8	59/104	7.02 (0.41)	1.32 (0.20)	2/2 (0.70)	3/4 (0.77)	5/6 (0.60)	10/11 (0.68)	29/26 (0.59)
SCORE + cg06644428	614.44; 10; <.0001	634.44	0.09	0.783 (0.723, 0.842)<br>0.738 (0.657, 0.801)[b]	10/12	57/12	2.89 (0.77)	1.19 (0.09)	2/1 (0.65)	3/3 (0.82)	2/6 (0.10)	12/11 (0.68)	30/27 (0.62)
SCORE + cg01940273	615.45; 10; <.0001	635.45	0.13	0.786 (0.727, 0.846)<br>0.743 (0.660, 0.804)[b]	10/8	74/120	9.17 (0.30)	0.60 (0.30)	2/1 (0.64)	3/3 (0.80)	1/6 (0.03)	14/11 (0.37)	29/27 (0.66)
SCORE + cg05575921 + cg06126421 + cg03636183	596.72; 12; <.0001	620.72	0.0003	0.817 (0.759, 0.876)<br>0.773 (0.684, 0.837)[b]	29/3	170/60	40.12 (0.0004)	5.65 (<.0001)	1/1 (0.93)	2/3 (0.64)	6/5 (0.75)	7/10 (0.32)	33/29 (0.55)
SCORE + cg05575921 + cg06126421 + cg21566642	596.45; 12; <.0001	620.45	0.0003	0.821 (0.764, 0.877)<br>0.774 (0.687, 0.838)[b]	28/3	179/59	37.70 (0.001)	5.56 (<.0001)	2/1 (0.39)	0/3 (0.09)	6/5 (0.77)	6/10 (0.17)	35/29 (0.30)
SCORE + cg05575921 + cg06126421 + cg05951221	595.58; 12; <.0001	619.58	0.0002	0.822 (0.764, 0.880)<br>0.778 (0.691, 0.843)[b]	31/3	180/66	44.53 (0.0002)	5.63 (<.0001)	1/1 (1.00)	3/3 (0.86)	4/5 (0.63)	7/10 (0.31)	34/29 (0.46)
SCORE + 5 CpGs[c]	598.25; 14; <.0001	617.25	<.0001	0.815 (0.754, 0.876)<br>0.771 (0.684, 0.840)[b]	30/4	174/66	41.08 (0.0007)	8.34 (<.0001)	1/1 (0.84)	4/2 (0.28)	3/5 (0.45)	9/10 (0.74)	32/31 (0.88)
SCORE + 9 CpGs[d]	586.45; 18; <.0001	622.45	0.0003	0.817 (0.756, 0.879)<br>0.754 (0.660, 0.822)[b]	31/5	173/72	41.83 (0.0007)	9.16 (<.0001)	1/1 (0.82)	4/2 (0.23)	4/5 (0.81)	7/10 (0.39)	33/32 (0.83)

Abbreviation: AIC, Akaike's information criterion; CI, confidence interval; IDI, Integrated discrimination improvement; LOG L, log-likelihood; LR, likelihood ratio; n_{exp}, number of expected events; n_{obs}, number of observed events; NRI, net reclassification improvement; Ref., reference; SCORE, Systematic Coronary Risk Evaluation chart: age, sex, systolic blood pressure, current smoking, and total cholesterol. [a]Comparison of SCORE+methylation-model with SCORE-model by likelihood ratio test. [b]Optimism-corrected Harrell's C statistics (95% CI) by .632 bootstrap. [c]5 CpGs: cg05575921 + cg06126421 + cg03636183 + cg21566642 + cg05951221; [d]9CpGs: cg05575921 + cg06126421 + cg03636183 + cg21566642 + cg05951221 + cg23576855 + cg21161138 + cg06644428 + cg01940273.

Table S7. Evaluation of the SCORE and methylation intensity in prediction of fatal cardiovascular disease (excluding participants with cardiovascular disease at baseline n=216)

Characteristics	SCORE	SCORE + cg05575921	SCORE + cg06126421	SCORE + cg05575921 cg06126421
Overall model fit				
-2 LOG L; df; p-value	243.95; 5; 003	228.96; 6; <.0001	230.93; 6; <.0001	227.01; 7; <.0001
AIC	253.95	240.96	242.93	241.01
LR test p-value[a]	---	0.0001	0.0003	0.0002
Harrell's C statistics (95% CI)	0.777 (0.694, 0.859)	0.842 (0.765, 0.918)	0.838 (0.766, 0.911)	0.862 (0.791, 0.933)
Optimism-corrected Harrell's C statistics (95% CI)	0.728 (0.617, 0.817)	0.810 (0.717, 0.887)	0.802 (0.705, 0.900)	0.817 (0.725, 0.900)
Reclassification of				
Cases, n_{up}/n_{down}	Ref.	7/2	7/1	8/2
Controls, n_{up}/n_{down}	Ref.	47/57	53/55	44/58
NRI % (p-value)	Ref.	26.37 (0.08)	30.27 (0.03)	31.92 (0.04)
IDI % (p-value)	Ref.	3.34 (0.06)	2.31 (0.03)	2.82 (0.01)
Calibration				
n_{obs}/n_{exp}(p-value)				
Quintile 1	0/1 (0.40)	0/0 (0.56)	0/0 (0.53)	0/0 (0.56)
Quintile 2	2/2(0.73)	1/1 (0.96)	0/1 (0.33)	1/1 (0.93)
Quintile 3	0/3 (0.10)	1/2 (0.51)	3/2 (0.38)	1/2 (0.55)
Quintile 4	8/4 (0.09)	2/4 (0.34)	3/4 (0.62)	2/4 (0.32)
Quintile 5	10/11 (0.85)	16/13 (0.39)	14/13 (0.75)	16/13 (0.40)

Abbreviation: AIC, Akaike's information criterion; CI, confidence interval; IDI, Integrated discrimination improvement; LOG L, log-likelihood; LR, likelihood ratio; n_{exp}, number of expected events; n_{obs}, number of observed events; NRI, net reclassification improvement; Ref., reference; SCORE, Systematic Coronary Risk Evaluation chart: age, sex, systolic blood pressure, current smoking and total cholesterol. [a]Comparison of SCORE+methylation-model with SCORE-model by likelihood ratio test.

Figure S1. Dose-response relationships between smoking behavior and methylation intensity

A. Dose-response relationships between current intensity of smoking and methylation intensity (never and former smokers were defined as reference with current smoking intensity = 0)

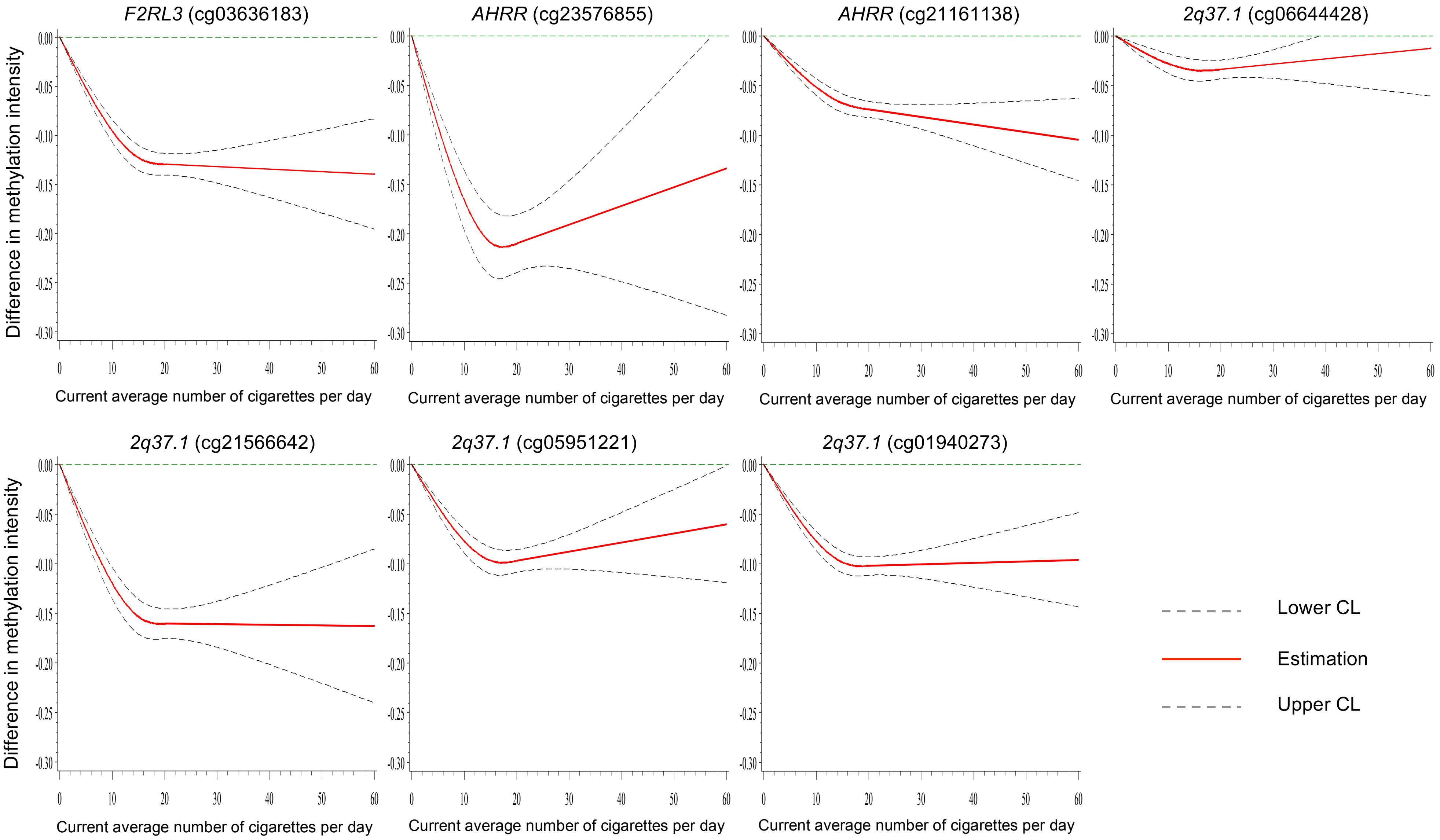

B. Dose-response relationships between cumulative dose of smoking and methylation intensity(never smokers were defined as reference with pack-years = 0)

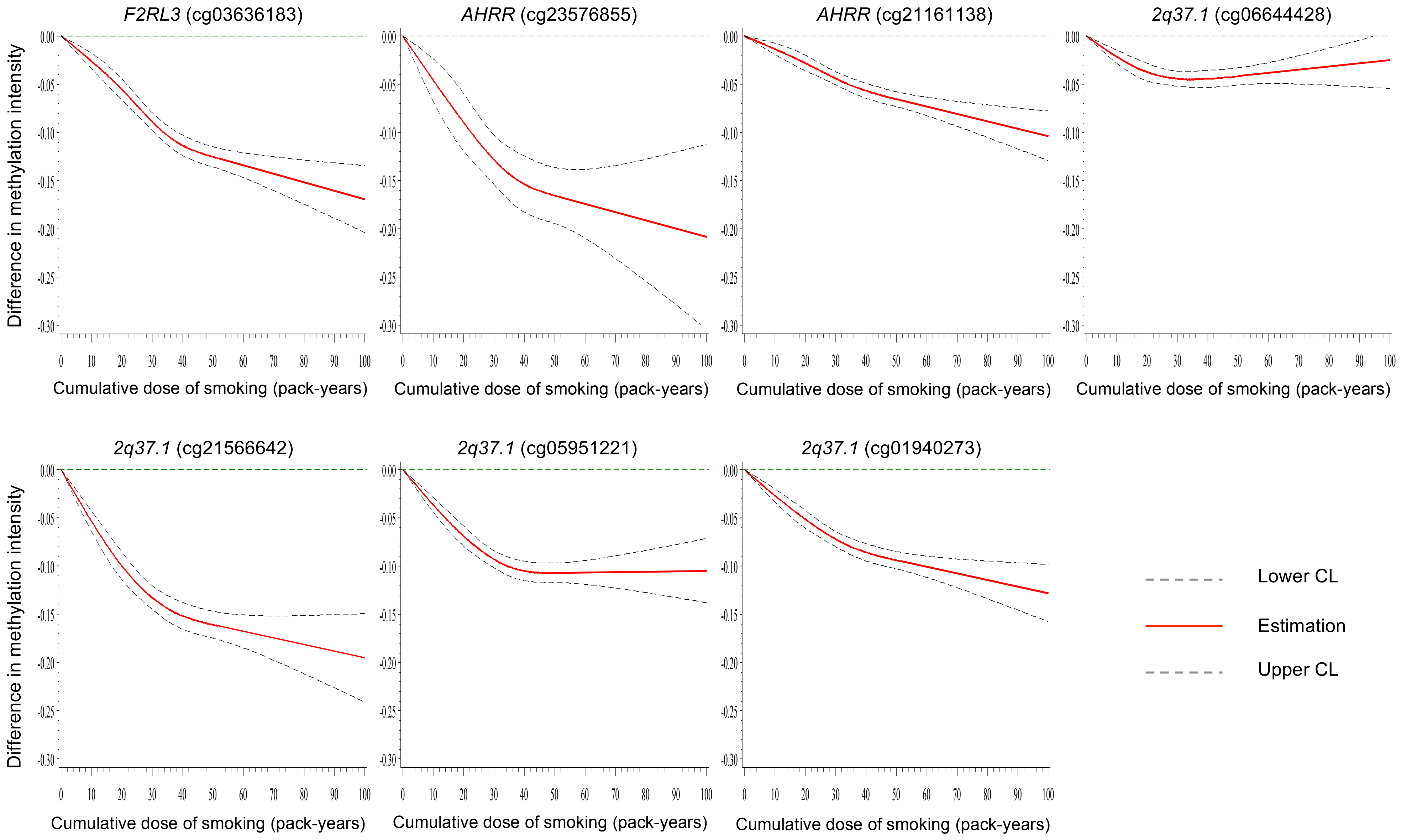

C. Dose-response relationships between time since cessation of smoking and methylation intensity among former smokers(current smokers were defined as reference with time since cessation = 0)

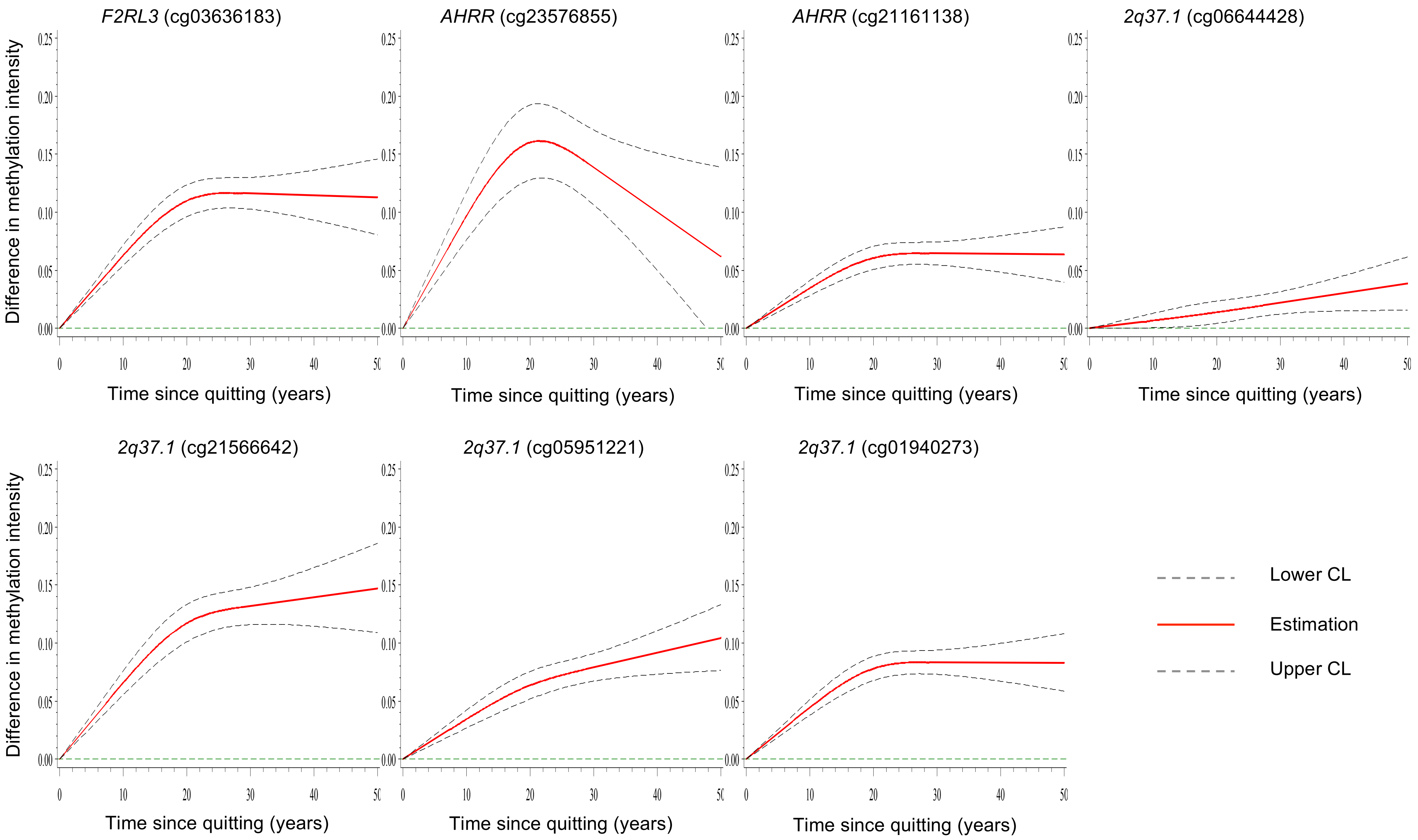

Figure S2. Kaplan-Meier estimates of survival by methylation quartiles

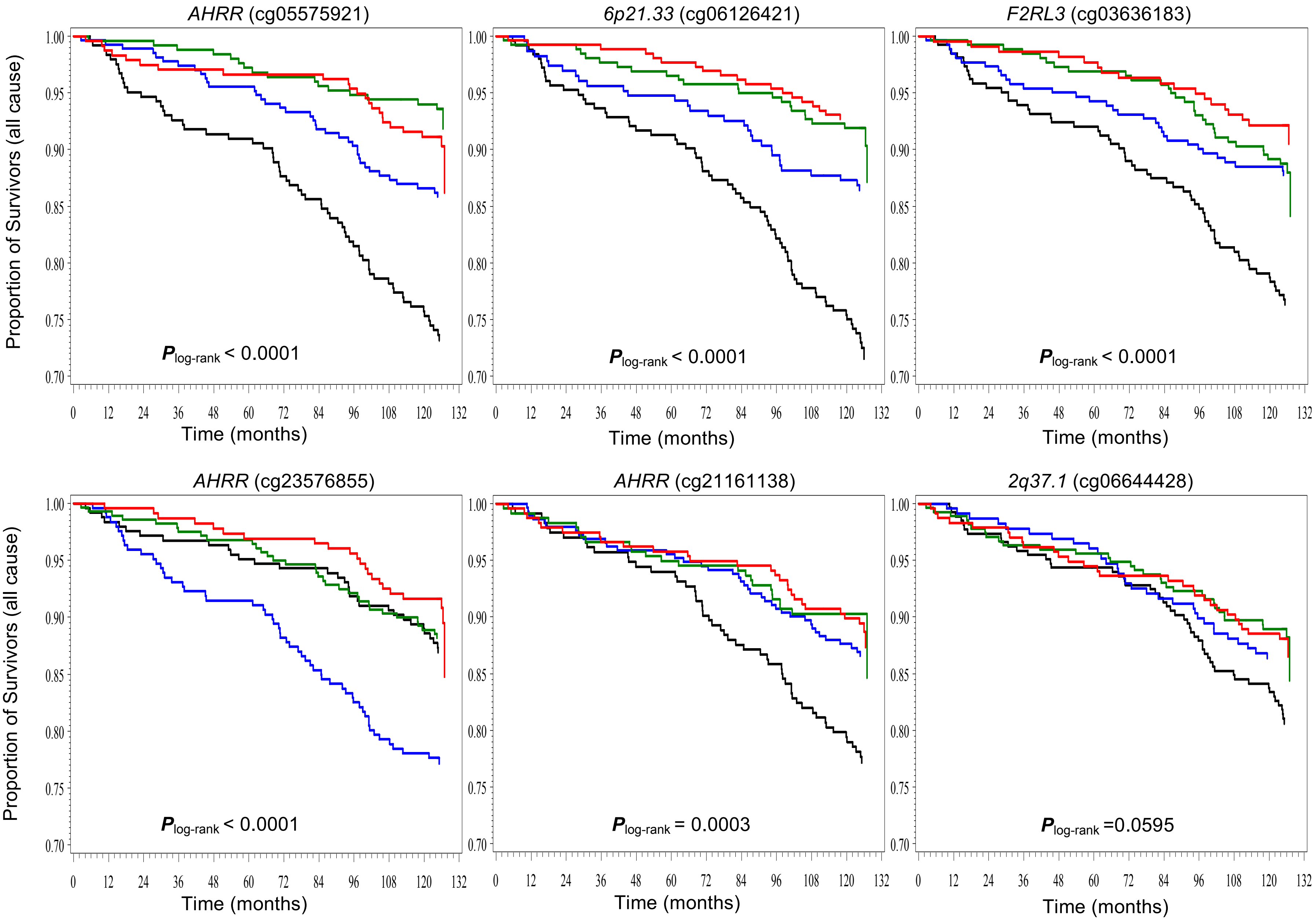

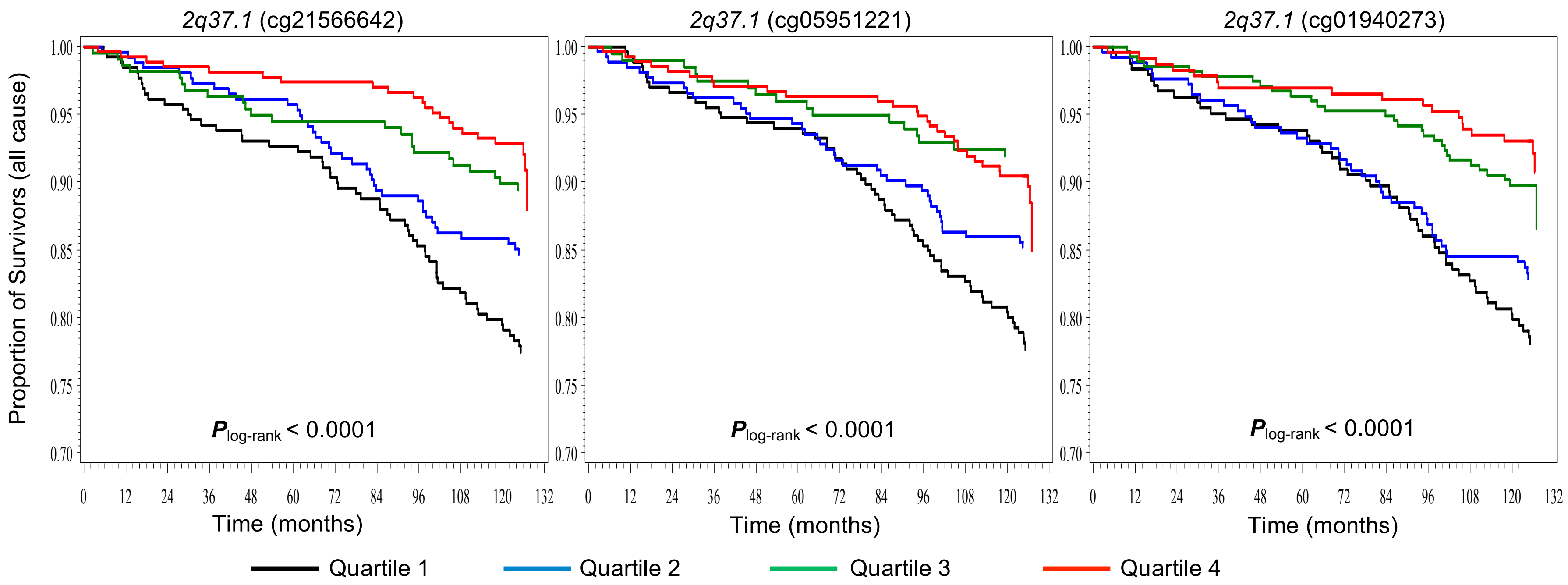

2q37.1 (cg21566642)
2q37.1 (cg05951221)
2q37.1 (cg01940273)
Proportion of Survivors (all cause)
1.00
0.95
0.90
0.85
0.80
0.75
0.70
$P_{log-rank}$ < 0.0001
0
12
24
36
48
60
72
84
96
108
120
132
Time (months)
Quartile 1
Quartile 2
Quartile 3
Quartile 4
